# Five New Secondary Metabolites Produced by a Marine-Associated Fungus, *Daldinia eschscholzii*

**DOI:** 10.3390/md12115563

**Published:** 2014-11-20

**Authors:** Zheng-Xi Hu, Yong-Bo Xue, Xiao-Bin Bi, Jin-Wen Zhang, Zeng-Wei Luo, Xiao-Nian Li, Guang-Min Yao, Jian-Ping Wang, Yong-Hui Zhang

**Affiliations:** 1 Hubei Key Laboratory of Natural Medicinal Chemistry and Resource Evaluation, School of Pharmacy, Tongji Medical College, Huazhong University of Science and Technology, Wuhan 430030, China; E-Mails: hzx616@126.com (Z.-X.H.); yongboxue@mail.hust.edu.cn (Y.-B.X.); bxbin8353@163.com (X.-B.B.); luozengwei@gmail.com (Z.-W.L.); gyap@mail.hust.edu.cn (G.-M.Y.); 2 Tongji Hospital Affiliated to Tongji Medical College, Huazhong University of Science and Technology, Wuhan 430030, China; E-Mail: tjzhangjinwen@163.com; 3 State Key Laboratory of Phytochemistry and Plant Resources in West China, Kunming Institute of Botany, Chinese Academy of Sciences, Kunming 650204, China; E-Mail: lixiaonian@mail.kib.ac.cn

**Keywords:** marine-associated fungus, *Daldinia eschscholzii*, secondary metabolites, hydrolysis, GC analysis, X-ray diffraction analysis

## Abstract

Five new compounds, including a benzopyran ribonic glycoside, daldiniside A (**1**), two isocoumarin ribonic glycosides, daldinisides B (**2**) and C (**3**), and two alkaloids, 1-(3-indolyl)-2*R*,3-dihydroxypropan-1-one (**4**) and 3-ethyl-2,5-pyrazinedipropanoic acid (**5**), along with five known compounds (**6**–**10**), were isolated from the EtOAc extract of the marine-associated fungus, *Daldinia eschscholzii*. Their structures were elucidated by extensive physicochemical and spectroscopic properties, besides comparison with literature data. The absolute configurations of compounds **1**–**3** were corroborated by chemical transformation, GC analysis and X-ray crystallographic analysis. Meanwhile, the absolute configuration of compound **4** and the planar structure of compound **6** were also determined based on the X-ray diffraction analysis. The cytotoxicity of compounds **1**–**10**, antifungal and anti-HIV activities of compounds **1**–**5** and the *in vitro* assay for glucose consumption of compounds **1**–**3** were done in the anti-diabetic model, whereas none showed obvious activity.

## 1. Introduction

Marine fungi are known as a rich source of structurally diverse and biologically active secondary metabolites, including polyketides, steroids, terpenes and alkaloids. Nevertheless, the potential chemical investigations on marine fungi are limited. In recent decades, bioactive natural products obtained from the marine-derived fungi have attracted the rising attention of organic chemists for discovering new drugs [1].

It was amazing that slight variations of traditional cultivation conditions, such as media compositions, temperature, aeration or the shape of the culturing flask, might lead to the discovery of various types of new natural products by microorganism [2]. As was reported, *Daldinia eschscholzii* was well-known to produce abundant polyketides as a mantis-associated fungus [3,4], which motivated us to investigate the secondary metabolites produced by the marine-associated fungus, *D. eschscholzii*. As part of our ongoing research for structurally unique and bioactive natural products from the *D. eschscholzii*, we obtained a new benzopyran ribonic glycoside (**1**), two new isocoumarin ribonic glycosides (**2** and **3**) and two new alkaloids (**4** and **5**), together with five known derivatives (**6**–**10**) from the scaled-up fermentation of the *D. eschscholzii*. Herein, we describe the isolation, structural elucidation and biological evaluations of these compounds.

## 2. Results and Discussion

### Chemical Structure Elucidation

**Figure 1:**
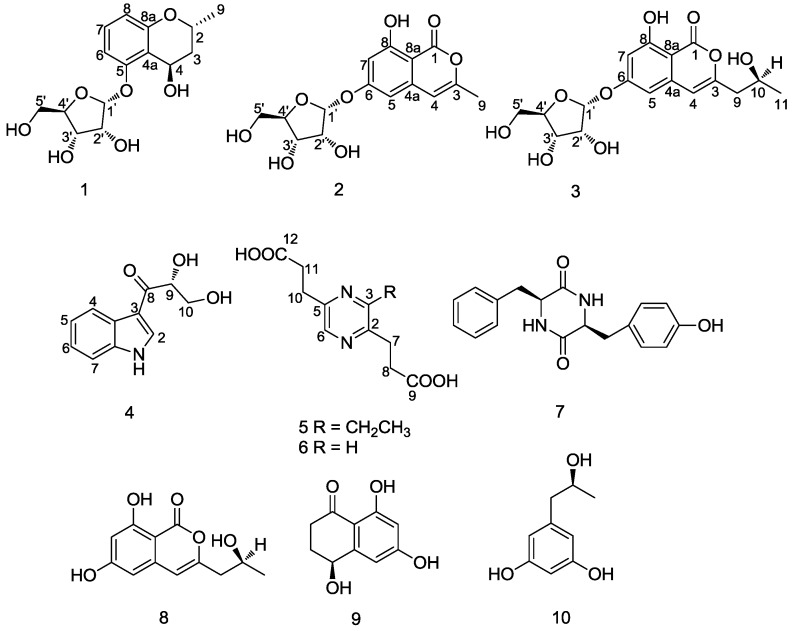
Structures of compounds **1**–**10**.

The EtOAc extract of the solid medium of *D.* *eschscholzii* was subjected to extensive chromatographic separations over silica gel CC, RP-C_18_ silica gel CC, Sephadex LH-20 and semi-preparative HPLC to yield a new benzopyran ribonic glycoside, daldiniside A (**1**), two new isocoumarin ribonic glycosides, daldinisides B (**2**) and C (**3**), and two new alkaloids (**4** and **5**), along with five known compounds, 2,5-pyrazinedipropanoic acid (**6**) [5], cyclo-(Phe-Tyr) (**7**) [6], de-*O*-methyldiaporthin (**8**) [7], 4,6,8-trihydroxy-3,4-dihydronaphthalen-1(2*H*)-one (**9**) [8] and orcinotriol (**10**) [9], as shown in Figure 1.

**Table 1:** ^1^H (400 MHz) and ^13^C (100 MHz) NMR data for compounds **1**–**3**.

NO.	1 ^a^		2 ^a^		3 ^b^	
	δ_H_ (*J* in Hz)	δ_C_	δ_H_ (*J* in Hz)	δ_C_	δ_H_ (*J* in Hz)	δ_C_
1				167.8		167.0
2	4.21, m	68.8				
3	1.68, ddd (4.1,12.0, 14.4) 2.01, dt (1.9, 14.4)	38.9		155.9		156.7
4	5.04, dd (1.9, 4.1)	60.1	6.31, s	105.8	6.40, s	106.7
4a		116.1		141.2		140.5
5		158.6	6.58, d (1.8)	104.5	6.70, d (2.1)	104.3
6	6.72, d (8.2)	107.9		166.1		165.9
7	7.12, t (8.2)	130.5	6.62, d (1.8)	103.9	6.89, d (2.1)	103.7
8	6.49, d (8.2)	112.0		164.5		164.1
8a		157.4		101.4		101.5
9	1.40, d (6.3)	21.6	2.22, s	19.4	2.67, dd (4.9, 14.3) 2.74, dd (7.8, 14.3)	44.3
10					4.45, m	65.4
11					1.40, d (6.2)	24.4
1′	5.70, d (4.5)	103.4	5.74, d (4.4)	101.8	6.17, d (4.2)	102.1
2′	4.23, dd (4.5, 6.4)	73.9	4.24, dd (4.4, 6.2)	73.6	4.87, m	74.1
3′	4.10, dd (2.6, 6.4)	71.4	4.12, dd (3.0, 6.2)	71.2	4.85, m	71.3
4′	4.20, m	88.5	4.15, m	88.3	4.88, m	89.0
5′	3.64, dd (3.9, 12.1) 3.69, dd (3.6, 12.1)	63.5	3.65, dd (3.8, 12.1) 3.72, dd (3.3, 12.1)	63.3	4.12, dd (3.8, 12.0) 4.17, dd (3.7, 12.0)	63.4

^a^ Measured in CD_3_OD; ^b^ measured in C_5_D_5_N.

Compound **1** was obtained as colorless crystal. Its molecular formula was determined as C_15_H_20_O_7_ by HRESIMS at *m/z* 335.1097 [M + Na]^+^ (calcd. for C_15_H_20_O_7_Na, 335.1107), indicating the presence of six degrees of unsaturation. The IR spectrum of **1** showed absorptions of hydroxyl (3423 cm^−1^) and aromatic (1611, 1588 and 1472 cm^−1^) functionalities. The ^1^H-NMR spectrum of **1** (Table 1) showed signals at δ_H_ 6.72 (1H, d, *J* = 8.2 Hz), 7.12 (1H, t, *J* = 8.2 Hz) and 6.49 (1H, d, *J* = 8.2 Hz), ascribed to one set of the typical 1,2,3-trisubstituted aromatic ring. Additionally, the ^1^H NMR spectrum of **1** also revealed the signals of one methyl group (δ_H_ = 1.40, d, *J* = 6.3 Hz) and two oxygen-bearing methines at 5.70 (1H, d, *J* = 4.5 Hz) and 5.04 (1H, dd, *J* = 1.9, 4.1 Hz). The ^13^C-NMR spectrum showed one methyl, two methylenes (one oxygenated), nine methines (three aromatic and six oxygenated) and three aromatic quaternary carbons. Furthermore, a series of proton signals at δ_H_ 3.64–5.70 and their corresponding carbons at δ_C_ 63.5, 71.4, 73.9, 88.5 and 103.4 might suggest a pentose moiety.

**Figure 2:**
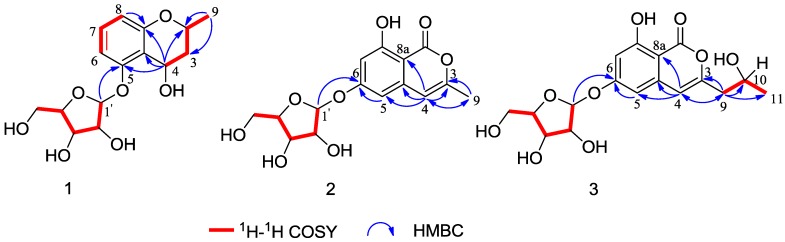
Selected ^1^H-^1^H COSY and HMBC correlations of compounds **1**–**3**.

Analysis of the key ^1^H-^1^H COSY and HMBC correlations (Figure 2) was used to establish the planar structure of **1**. In the HMBC spectrum, a diagnostic long-range correlation from the anomeric proton H-1′ to C-5 (δ_C_ 158.6) suggested that the sugar moiety was linked to the C-5 of aglycone. The remaining one degree of unsaturation, together with the ^1^H-^1^H COSY correlations of H-9/H-2, H-2/H-3 and H-3/H-4 and the HMBC correlations from H-9 to C-2, C-3 and from H-4 to C-2, C-4a, C-5 and C-8a, indicating that a pyranoid ring was linked to C-4a and C-8a, and the methyl and hydroxyl groups were located at C-2 and C-4, respectively. Thus, the planar structure of **1** was established.

**Figure 3:**
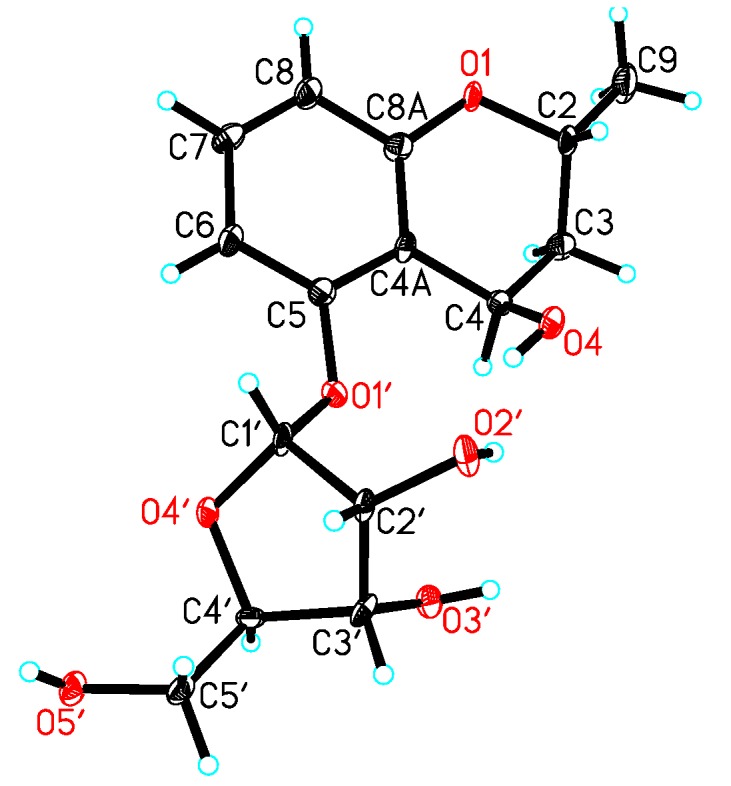
X-ray structure of compound **1**.

Acid hydrolysis of **1** gave the sugar motif, and then, it was unambiguously established as d-ribose by chemical transformation and GC analysis. The coupling constant of the anomeric proton at δ_H_ 5.70 (H-1′, d, *J* = 4.5 Hz) in the ^1^H NMR spectrum of **1** indicated the d-ribose unit to be in the α-configuration [10]. In the NOESY experiment, the correlations of H-2/H-4 or H-9/H-4 were not observed; Thus, it was difficult to determine the configurations at C-2 and C-4. Fortunately, we obtained the crystal of **1**, and a single crystal X-ray diffraction experiment was carried out with Cu Kα radiation (Figure 3), allowing an explicit assignment of the absolute structure as 2*R* and 4*R*. Hence, the absolute configuration of **1** was elucidated and named daldiniside A.

Compound **2** was isolated as a yellowish solid with the molecular formula C_15_H_16_O_8_, as deduced by the HRESIMS result ([M + Na]^+^ at *m*/*z* 347.0731, calcd. for C_15_H_16_O_8_Na, 347.0743). The presence of hydroxyl, carbonyl and double bond groups were shown by IR absorption bands at 3429, 1689 and 1573 cm^−1^, respectively. The α-d-ribose group of **2** was confirmed by NMR experiment (Table 1) and acid hydrolysis. The attachment of the α-d-ribose at C-6 was determined on the basis of the HMBC correlation from H-1′ (δ_H_ 5.74) to C-6 (δ_C_ 166.1). Apart from the signals of the sugar moiety, the ^1^H NMR spectrum showed proton signals at δ_H_ 6.58 (1H, d, *J* = 1.8 Hz) and 6.62 (1H, d, *J* = 1.8 Hz), indicating the presence of a 1,2,3,5-tetrasubstituted aromatic ring. This structural assignment was further established by the HMBC correlations from H-5 to C-6, C-7 and C-8a, and from H-7 to C-5, C-6, C-8 and C-8a. The ^13^C NMR spectrum showed one ester carbon signal at C-1 (δ_C_ 167.8), one olefinic carbon signal at C-4 (δ_C_ 105.8) and one methyl carbon signal at C-9 (δ_C_ 19.4). The HMBC correlations (Figure 2) from H-9 to C-3, C-4 and from H-4 to C-3, C-4a, C-5, C-8a and C-9 suggested that there existed an isocoumarin unit, in which the hydroxyl and methyl groups were located at C-8 and C-3, respectively. Thus, the structure of **2** was established, namely, daldiniside B.

Compound **3** was determined to be C_17_H_20_O_9_ by the HRESIMS data, which showed a molecular ion at *m/z* 391.0994 [M + Na]^+^ (calcd. for C_17_H_20_O_9_Na, 391.1005). The NMR data of **3 **were very similar to those of **2** (Table 1), suggesting that they shared the same basic skeleton. Moreover, the signals for a methylene at C-9 (δ_C_ 44.3), an oxygenated methine at C-10 (δ_C_ 65.4) and a methyl at C-11 (δ_C_ 24.4) were observed in the ^13^C NMR of **3**, from which we deduced that a -CH_2_(9)-CH(10)OH-CH_3_(11)- group in **3** replaced a -CH_3_ group in **2**. Hence, the planar structure of **3** was determined (Figure 2). To ascertain the absolute configuration at C-10, an acid hydrolysis experiment was carried out. By the chemical transformation and GC analysis, we established the sugar moiety to be α-d-ribose. In addition, the CHCl_3_ layer was evaporated to dryness, and the NMR data of the residual compound was identical to de-*O*-methyldiaporthin (${\left[\text{α}\right]}_{\text{D}}^{20}$: +20.0, *c* 0.09, MeOH). Therefore, the absolute configuration of **3** was established, namely daldiniside C.

**Figure 4:**
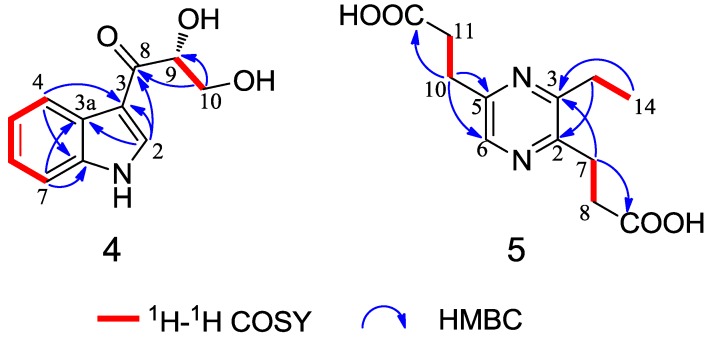
Selected ^1^H-^1^H COSY and HMBC correlations of compounds **4** and **5**.

**Figure 5:**
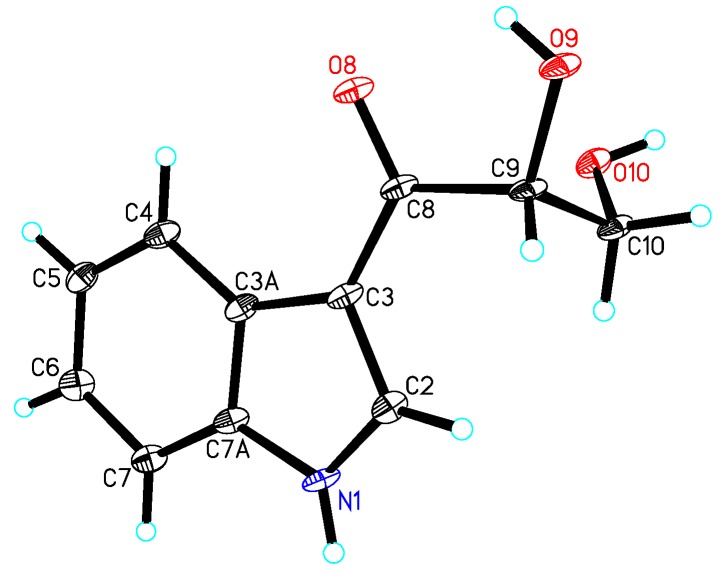
X-ray structure of compound **4**.

Compound **4** was obtained as a colorless crystal. The molecular formula C_11_H_11_NO_3 _was determined upon analysis of the HRESIMS peak at *m/z* 228.0628 [M + Na]^+^ (calcd. for C_11_H_11_NO_3_Na, 228.0637). UV absorption bands at 210, 243, 257 and 300 nm and IR absorption bands at 3394, 3325 and 1607 cm^−1^ implied the presence of amine, hydroxy and conjugated carbonyl functionalities. In the ^1^H NMR spectrum (Table 2), the signals at δ_H_ 12.03 (1H, s), 8.21 (1H, dd, *J* = 2.0, 6.6 Hz), 7.19 (1H, m), 7.23 (1H, m) and 7.49 (1H, dd, *J* = 1.7, 6.8 Hz) indicated the presence of an unsubstituted indole aromatic ring, which was inferred by the ^1^H-^1^H COSY correlations of H-4/H-5, H-5/H-6 and H-6/H-7 (Figure 4). The -CO(8)-CH(9)OH-CH_2_(10)OH- subunit was established by analysis of the ^1^H-^1^H COSY correlation of H-9/H-10 and HMBC correlation from H-10 to C-8 and C-9 and linked to the indole moiety by C-3, determined by the HMBC correlations from H-2 to C-3 and C-8. The configuration at C-9 was unequivocally established to be *R* by the single-crystal X-ray diffraction using Cu Kα radiation (Figure 5). Consequently, the absolute configuration of **4** was established and named 1-(3-indolyl)-2*R*,3-dihydroxypropan-1-one.

**Table 2:** ^1^H and ^13^C NMR data for compounds **4**–**6**.

NO.	4 ^c^		5 ^d^		6 ^c^	
	δ_H_ (*J* in Hz)	δ_C_	δ_H_ (*J* in Hz)	δ_C_	δ_H_ (*J* in Hz)	δ_C_
1	12.03, s					
2	8.41, s	134.8		152.2		153.0
3		114.1		157.2	8.44, d (4.1)	143.3
3a		125.9				
4	8.21, dd (2.0, 6.6)	121.4				
5	7.19, m	121.9		153.9		153.0
6	7.23, m	122.9	8.25, s	141.7	8.44, d (4.1)	143.3
7	7.49, dd (1.7, 6.8)	112.2	3.10, t (7.2)	29.3	2.96, t (7.3)	29.2
7a		136.3				
8		195.5	2.79, t (7.2)	32.9	2.67, t (7.3)	32.2
9	4.69, t (4.5)	75.8		177.1		173.8
10	3.63, dd (5.5, 11.1) 3.71, dd (4.5, 11.1)	65.3	3.04, t (7.2)	30.7	2.96, t (7.3)	29.2
11			2.76, t (7.2)	33.9	2.67, t (7.3)	32.2
12				176.9		173.8
13			2.88, q (7.5)	28.3		
14			1.29, t (7.5)	13.1		

^c^ Measured in DMSO-*d*_6_ on a Bruker AM-400 spectrometer; ^d^ measured in CD_3_OD on a Bruker DRX-600 spectrometer.

**Figure 6:**
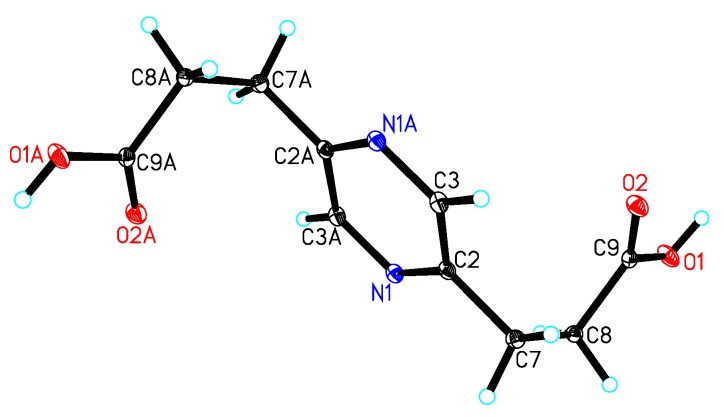
X-ray structure of compound **6**.

Compound **5** was isolated as a yellow oil with the molecular formula C_10_H_12_N_2_O_4_ as determined by the HRESIMS peak at *m/z* 275.0998 [M + Na]^+^ (calcd. for C_12_H_16_N_2_O_4_Na, 275.1008). In the 2D NMR spectra of **5** (Figure 4), the ^1^H-^1^H COSY correlations of H-7/H-8 and H-10/H-11 and the HMBC correlations from H-8 to C-2 and C-9, from H-11 to C-5 and C-12 and from H-6 to C-2 and C-5 indicated the existence of the 2,5-pyrazinedipropanoic acid group. An additional ethyl moiety was located at C-3 by the ^1^H-^1^H COSY correlation of H-13/H-14 and a long-rang HMBC correlation from H-14 to C-3. Thus, the structure of **5** was established and named 3-ethyl-2,5-pyrazinedipropanoic acid, whose signals were similar to **6** (Table 2), confirmed by a single-crystal X-ray diffraction using Mo Kα radiation (Figure 6).

## 3. Experimental Section

### 3.1. General Experimental Procedures

UV spectra were measured on a Varian Cary 50 spectrophotometer or a Shimadzu UV-2401A spectrophotometer. Optical rotations were recorded on a Perkin-Elmer PE-341LC polarimeter. IR spectra were determined on a Bruker Vertex 70 FT-IR spectrophotometer. The ^1^H, ^13^C, and 2D NMR spectroscopic data were recorded on Bruker AM-400 and DRX-600 spectrometers using TMS as the internal standard. HRESIMS data were acquired using an APIQSTAR Pulsar spectrometer. X-ray data were collected using a Bruker APEX DUO diffractometer. Column chromatography was performed on silica gel (100–200 mesh and 200–300 mesh, Qingdao Marine Chemical Inc., Qingdao, China), RP-C_18_ silica gel (50 μm, YMC, Kyoto, Japan) and Sephadex LH-20 (Pharmacia Biotech AB, Uppsala, Sweden). Semi-preparative HPLC was conducted on an Agilent 1100 liquid chromatography with a YMC-Pack ODS-A (10 × 250 mm, 5μm, YMC Co., Ltd., Kyoto, Japan) column. GC analysis was performed with an Agilent Technologies 6890N gas chromatography system. Solvents were distilled prior to use, and spectroscopic grade solvents were used. TLC was performed with silica gel 60 F254 (Yantai Chemical Industry Research Institute, Yantai, China) and RP-C_18_ F254 plates (Merck, Darmstadt, Germany). Peptone (produced by protamine with enzymatic hydrolysis and drying into a pale yellow powder) was purchased from Beijing Shuangxuan Microbial Medium Plant (Product ID: 02-31A, Specification: BR).

### 3.2. Fungal Material and Fermentation

The strain of fungus *D. eschscholzii* was isolated from the branches of *Scaevola sericea* Vahl, collected from the mangrove forest nature reserve in Haikou, Hainan province, China. The fungus was identified by sequence analysis of the ITS region of its rDNA, as described previously [11], and the sequence data have been deposited in NCBI with Accession Number FJ624265. A voucher specimen (MCCC 3J00088) was deposited in a public collection, the Marine Culture Collection of China, MCCC. All of the information and strains collected can be shared at the website http://www.mccc.org.cn/ and the collection center.

The strain *D. eschscholzii* was cultivated on a potato dextrose agar (PDA) plate at 25 °C for 15 days. The agar was cut into pieces (0.5 × 0.5 cm^2^) and inoculated into 100 × 500 mL Erlenmeyer (composition: normal rice (100 g), peptone (0.5 g), in distilled water (100 mL)) at 28 °C for 21 days under static conditions.

### 3.3. Extraction and Isolation

The fermented rice substrate was extracted four times with EtOAc (4 × 25 L) at room temperature. After concentration* in vacuo*, the total extract (145.0 g) was suspended in water and then extracted exhaustively with petroleum ether and EtOAc, respectively. The EtOAc organic phase was evaporated under reduced pressure to afford a crude extract (77.0 g), which was subjected to silica gel column chromatography (CC) with a CH_2_Cl_2_/CH_3_OH gradient system (1:0, 50:1, 25:1, 10:1, 6:1, 3:1 and 1:1, v/v, each 8 L) to obtain six main fractions (A−F).

Fraction C (CH_2_Cl_2_/CH_3_OH, 10:1; 8.6 g) was subjected to RP-C_18_ silica gel CC (CH_3_OH/H_2_O, 20:80 to 100:0, 12 L) to get five subfractions (C1–C5). Subfraction C2 (CH_3_OH/H_2_O, 40:60; 2.8 g) was subjected to Sephadex LH-20 (CH_3_OH, 1.2 L), then separated by silica gel CC eluted with CH_2_Cl_2_–CH_3_OH (50:1, v/v, 1.9 L) and by semi-preparative HPLC using CH_3_OH–H_2_O (2.5 mL/min, CH_3_OH:H_2_O = 50:50, v/v) and CH_3_CN–H_2_O (2.5 mL/min, CH_3_CN:H_2_O = 14:86, v/v) to yield **8** (7.4 mg, *t*_R_ = 22.0 min) and **9** (2.6 mg, *t*_R_ = 37.0 min), respectively. Subfraction C3 (CH_3_OH/H_2_O, 60:40; 1.2 g) was fractionated by Sephadex LH-20 CC with CH_2_Cl_2_/CH_3_OH (1:1, v/v, 650 mL), silica gel CC (CH_2_Cl_2_–CH_3_OH, 100:1, 1.1 L) and further purified by semi-preparative HPLC using CH_3_OH–H_2_O (2.5 mL/min, CH_3_OH:H_2_O = 60:40, v/v) to obtain **5** (12.3 mg, *t*_R_ = 11.7 min).

Fraction D (CH_2_Cl_2_/CH_3_OH, 6:1; 9.5 g) was subjected to RP-C_18_ silica gel CC eluted with CH_3_OH–H_2_O (20:80 to 100:0, 16 L) to afford five subfractions (D1–D5). Subfraction D1 (CH_3_OH/H_2_O, 20:80; 2.1 g) was crystallized in CH_3_OH to yield **6** (30.5 mg) and then chromatographed over Sephadex LH-20 with CH_2_Cl_2_/CH_3_OH (1:1, v/v, 1.3 L), followed by RP-C_18_ silica gel CC eluted with CH_3_OH–H_2_O (10:90 to 20:80, v/v, 4 L) to yield four fractions (D1.1–D1.4). Fraction D1.2 (CH_3_OH/H_2_O, 10:90; 505.0 mg) was subjected to silica gel CC (CH_2_Cl_2_–CH_3_OH, 75:1, 750 mL) and purified by semi-preparative HPLC (2.5 mL/min, CH_3_CN/H_2_O, 13:87) to yield **10** (14.4 mg, *t*_R_ = 14.7 min). Fraction D1.3 (CH_3_OH/H_2_O, 15:85; 75.0 mg) was subjected to silica gel CC (CH_2_Cl_2_–CH_3_OH, 50:1, 450 mL) and further purified by semi-preparative HPLC (2.5 mL/min, CH_3_OH/H_2_O, 20:80) to get **4** (11.4 mg, *t*_R_ = 40.0 min). Subfraction D2 (CH_3_OH/H_2_O, 40:60; 2.2 g) was fractionated by Sephadex LH-20 CC with CH_3_OH (1.2 L), followed by silica gel CC eluted with CH_2_Cl_2_–CH_3_OH (50:0 to 20:1, v/v, 1.8 L) to obtain three fractions (D2.1–D2.3). Fraction D2.3 (CH_2_Cl_2_/CH_3_OH, 20:1; 300.0 mg) was purified by semi-preparative HPLC (2.5 mL/min, CH_3_OH/H_2_O, 30:70) to obtain **3** (250.0 mg, *t*_R_ = 37.5 min). Fraction D2.2 (CH_2_Cl_2_/CH_3_OH, 30:1; 1.2 g) was successively purified by semi-preparative HPLC using CH_3_CN−H_2_O (2.5 mL/min, CH_3_CN:H_2_O = 30:70, v/v) to yield **2** (23.0 mg, *t*_R_ = 11.0 min) and CH_3_CN–H_2_O (2.5 mL/min, CH_3_CN:H_2_O = 17:83, v/v) to yield **1** (6.5 mg, *t*_R_ = 36.2 min) and **7** (6.0 mg, *t*_R_ = 39.0 min), respectively.

Daldiniside A (**1**): Colorless crystal; ${\left[\text{α}\right]}_{\text{D}}^{20}$: +106.3 (*c* = 0.16 mg/mL, MeOH); UV (MeOH) λ_max_ (log ε): 206 (4.41), 225 (3.78) and 282 (3.18) nm; IR (KBr) ν_max_: 3423, 2926, 1611, 1588, 1472, 1263, 1244, 1126, 1088, 1044, 1026 cm^−1^; ^1^H and ^13^C NMR data, see Table 1; HRESIMS *m/z* 335.1097 [M + Na]^+^ (calcd. for C_15_H_20_O_7_Na, 335.1107).

Daldiniside B (**2**): Yellowish powder; ${\left[\text{α}\right]}_{\text{D}}^{20}$: +14.1 (*c* = 0.67 mg/mL, MeOH); UV (MeOH) λ_max_ (log ε): 236 (4.56), 243 (4.57) and 329 (3.69) nm; IR (KBr) ν_max_: 3429, 2926, 1689, 1644, 1624, 1573, 1506, 1385, 1354, 1235, 1172, 1074, 1043, 694 cm^−1^; ^1^H and ^13^C NMR data, see Table 1; HRESIMS *m/z* 347.0731 [M + Na]^+^ (calcd. for C_15_H_16_O_8_Na, 347.0743).

Daldiniside C (**3**): Yellowish powder; ${\left[\text{α}\right]}_{\text{D}}^{20}$: +45.0 (*c* = 0.13 mg/mL, MeOH); UV (MeOH) λ_max_ (log ε): 203 (3.62), 244 (3.92) and 334 (3.06) nm; IR (KBr) ν_max_: 3427, 2924, 1686, 1626, 1505, 1384, 1240, 1171, 1127, 1083, 1045, 695 cm^−1^; ^1^H and ^13^C NMR data, see Table 1; HRESIMS *m/z* 391.0994 [M + Na]^+^ (calcd. for C_17_H_20_O_9_Na, 391.1005).

1-(3-indolyl)-2*R*,3-dihydroxypropan-1-one (**4**): Colorless crystal; ${\left[\text{α}\right]}_{\text{D}}^{20}$: +20.0 (*c* = 0.70 mg/mL, MeOH); UV (MeOH) λ_max_ (log ε): 210 (4.19), 243 (3.82), 257 (3.70) and 300 (3.82) nm; IR (KBr) ν_max_: 3394, 3325, 1607, 1520, 1442, 1156, 1090, 986, 742, 705 cm^−1^; ^1^H and ^13^C NMR data, see Table 2; HRESIMS *m/z* 228.0628 [M + Na]^+^ (calcd. for C_11_H_11_NO_3_Na, 228.0637).

3-ethyl-2,5-pyrazinedipropanoic acid (**5**): Yellow oil; UV (MeOH) λ_max_ (log ε): 210 (3.94), 243 (3.18) and 279 (3.86) nm; IR (KBr) ν_max_: 2975, 2935, 1714, 1452, 1392, 1251, 1177, 1122 cm^−1^; ^1^H and ^13^C NMR data, see Table 2; HRESIMS *m/z* 275.0998 [M + Na]^+^ (calcd. for C_12_H_16_N_2_O_4_Na, 275.1008) and *m/z* 253.1178 [M + H]^+^ (calcd. for C_12_H_17_N_2_O_4_, 253.1188).

### 3.4. X-ray Crystallographic Analysis

Crystal data for **1**: C_15_H_20_O_7_, *M* = 312.31, orthorhombic, *a* = 5.3212(2) Å, *b* = 10.3774(4) Å, *c* = 26.8183(9) Å, *α* = 90.00°, *β* = 90.00°, *γ* = 90.00°, *V* = 1480.91(9) Å^3^, *T* = 100(2) K, space group *P*212121, *Z* = 4, *μ*(Cu Kα) = 0.943 mm^−1^, 10,224 reflections measured, 2578 independent reflections (*R_int_* = 0.1191). The final *R_1_* values were 0.1218 (*I* > 2*σ*(*I*)). The final *wR*(*F*^2^) values were 0.3053 (*I* > 2*σ*(*I*)). The final *R_1_* values were 0.1580 (all data). The final *wR*(*F*^2^) values were 0.3760 (all data). The goodness of fit on *F*^2^ was 1.445. Flack parameter = 0.0 (7).

Crystal data for **4**: C_11_H_11_NO_3_, *M* = 205.21, monoclinic, *a* = 4.7449(5) Å, *b* = 5.4635(5) Å, *c* = 17.8653(16) Å, *α* = 90.00°, *β* = 95.820(5)°, *γ* = 90.00°, *V* = 460.75(8) Å^3^, *T* = 100(2) K, space group *P*21, *Z* = 2, *μ*(Cu Kα) = 0.903 mm^−1^, 3,878 reflections measured, 1,498 independent reflections (*R*_int_ = 0.0490). The final *R_1_* values were 0.0523 (*I* > 2*σ*(*I*)). The final *wR*(*F^2^*) values were 0.1467 (I > 2σ(I)). The final *R_1_* values were 0.0527 (all data). The final *wR*(*F^2^*) values were 0.1470 (all data). The goodness of fit on *F^2^* was 1.109. Flack parameter = −0.4(4). The Hooft parameter is 0.02(13) for 567 Bijvoet pairs.

Crystal data for **6**: C_10_H_12_N_2_O_4_, *M* = 224.22, monoclinic, *a* = 5.6215(9) Å, *b* = 13.275(2) Å, *c* = 7.0331(11) Å, *α* = 90.00°, *β* = 105.791(2)°, *γ* = 90.00°, *V* = 505.04(14) Å^3^, *T* = 100(2) K, space group *P*21*/n*, *Z* = 2, *μ*(Mo Kα) = 0.116 mm^−1^, 5,086 reflections measured, 1415 independent reflections (*R_int_* = 0.0238). The final *R_1_* values were 0.0368 (*I* > 2*σ*(*I*)). The final *wR*(*F*^2^) values were 0.0918 (*I* > 2*σ*(*I*)). The final *R_1_* values were 0.0380 (all data). The final *wR*(*F*^2^) values were 0.0926 (all data). The goodness of fit on *F*^2^ was 1.088.

The crystallographic data for **1** (deposition No. CCDC 989294), **4** (deposition No. CCDC 981181), and **6** (deposition No. CCDC 981180) have been deposited in the Cambridge Crystallographic Data Centre. Copies of the data can be obtained free of charge from the Cambridge Crystallographic Data Centre, 12 Union Road, Cambridge CB21EZ, UK (fax: +44-1223-336-033; or E-Mail: deposit@ccdc.cam.ac.uk).

### 3.5. Acid hydrolysis and GC Analysis of **1**−**3** and Determination of the Absolute Configuration of the Sugar Moiety

Compound **1** (1.2 mg) was hydrolyzed with 2 M aqueous CF_3_COOH (2.0 mL) at 90 °C for 6 h. The reaction mixture was evaporated to dryness; Then, the residue and l-cysteine methyl ester hydrochloride (2.5 mg) were dissolved in dry pyridine (1.0 mL) and kept at 65 °C for 2 h. The reaction mixture was dried, and then, trimethylsilylimidazole (0.2 mL) was added to the residue, followed by stirring at 65 °C for 1 h [12]. In the end, the resultant solution was extracted with water and *n*-hexane, and then, the organic phase was submitted to GC analysis by using an HP-5MS capillary column (30 m × 0.25 mm × 0.25 μm, Agilent, Shanghai, China); column temperature, 230 °C; injection temperature, 250 °C; detector FID, detector temperature, 250 °C. A peak at the retention time of 12.61 min for compound **1** was observed. When the corresponding ribose was prepared by the same reaction, the retention times of presilylated d-ribose and l-ribose were 12.66 and 14.09 min, respectively. Hence, the sugar in compound **1 **was determined to be d-ribose.

Compounds **2** (1.5 mg) and **3** (9.8 mg) were subjected to a similar treatment as compound **1**, and the retention times of ribose were 12.63 and 12.66 min, respectively. Therefore, the sugar in compounds **2** and **3** were determined to be d-ribose. In addition, the reaction mixture of compound **3** was diluted with H_2_O (1.5 mL) and extracted with CHCl_3_. The CHCl_3_ layer was dried to yield the aglycone, whose NMR and optical rotation data were identical to de-*O*-methyldiaporthin (${\left[\text{α}\right]}_{\text{D}}^{20}$: +20.0, *c* 0.09, MeOH) [7].

### 3.6. Biological Activities

The cytotoxicity of **1**–**10** against HL-60, SMMC-7721, A-549, MCF-7 and SW-480 was studied using the MTT method [13], and the results showed no obvious inhibitory activity toward the above cancer cells with IC_50_ > 40 μg/mL. In addition, compounds **1**–**5** were tested for antifungal activities against *Candida albicans* (ATCC32354 and ATCC10231) at a concentration of 128 μg/mL and anti-HIV activity according to the described method [14,15]; Unfortunately, none of the compounds exhibited significant activities. Otherwise, the *in vitro* assay for glucose consumption of compounds **1**–**3** was done in the anti-diabetic model with DMEM-induced 3T3 fibroblasts [16], whereas none showed obvious activity at the concentration of 20 μg/mL.

## 4. Conclusions

A new benzopyran glycoside, daldiniside A (**1**), two new isocoumarin glycosides, daldinisides B (**2**) and C (**3**), and two new alkaloids, 1-(3-indolyl)-2*R*,3-dihydroxypropan-1-one (**4**) and 3-ethyl-2,5-pyrazinedipropanoic acid (**5**), together with five known compounds, 2,5-pyrazinedipropanoic acid (**6**), cyclo-(Phe-Tyr) (**7**), de-*O*-methyldiaporthin (**8**), 4,6,8-trihydroxy-3,4-dihydronaphthalen-1(2*H*)-one (**9**) and orcinotriol (**10**), were discovered from the marine-associated fungus, *D. eschscholzii*. Natural products embodying the α-d-ribose moiety were quite scarce to be reported. To the best of our knowledge, compounds **2** and **3** were hitherto the first example of isocoumarins containing the α-d-ribose moiety in natural products. Interestingly, the nonenzymatic cyclic dimerization of 5-aminolevulinic acid (5-ALA) might be the key reaction to form a pyrazine nucleus, and a series of derivatives might lead to the formation of **5** and **6** [17].

## Supplementary Files

Supplementary File 1
