# Supplementary material for: Five New Secondary Metabolites Produced by a Marine-Associated Fungus, Daldinia eschscholzii
Source: Mar Drugs. 2014 Nov 20;12(11):5563–75. doi: 10.3390/md12115563 (PMC4245545; doi:10.3390/md12115563)
Supplement: Supplementary File 1 [file marinedrugs-12-05563-s001.pdf]

Supplementary Information

Figure S1 ^{1}H NMR spectrum of **1** at 400 MHz in CD_3OD

Figure S2 ^{13}C NMR spectrum of **1** at 100 MHz in CD_3OD

Figure S3 HSQC spectrum of **1** in CD_3OD

Figure S4 ^{1}H-^{1}H COSY spectrum of **1** in CD_3OD

Figure S5 HMBC spectrum of **1** in CD_3OD

Figure S6 HRESIMS spectrum of **1**

Figure S7 IR spectrum of **1**

Figure S8 UV spectrum of **1**

Figure S9 ^{1}H NMR spectrum of **2** at 400 MHz in CD_3OD

Figure S10 ^{13}C NMR spectrum of **2** at 100 MHz in CD_3OD

Figure S11 HSQC spectrum of **2** in CD_3OD

Figure S12 ^{1}H-^{1}H COSY spectrum of **2** in CD_3OD

Figure S13 HMBC spectrum of **2** in CD_3OD

Figure S14 HRESIMS spectrum of **2**

Figure S15 IR spectrum of **2**

Figure S16 UV spectrum of **2**

Figure S17 ^{1}H NMR spectrum of **3** at 400 MHz in C_5D_5N

Figure S18 ^{13}C NMR spectrum of **3** at 100 MHz in C_5D_5N

Figure S19 HSQC spectrum of **3** in C_5D_5N

Figure S20 ^{1}H-^{1}H COSY spectrum of **3** in C_5D_5N

Figure S21 HMBC spectrum of **3** in C_5D_5N

Figure S22 HRESIMS spectrum of **3**

Figure S23 IR spectrum of **3**

Figure S24 UV spectrum of **3**

Figure S25 ^{1}H NMR spectrum of **4** at 400 MHz in DMSO-d_6

Figure S26 ^{13}C NMR spectrum of **4** at 100 MHz in DMSO-d_6

Figure S27 HSQC spectrum of **4** in DMSO-d_6

Figure S28 ^{1}H-^{1}H COSY spectrum of **4** in DMSO-d_6

Figure S29 HMBC spectrum of **4** in DMSO-d_6

Figure S30 HRESIMS spectrum of **4**

Figure S31 IR spectrum of **4**

Figure S32 UV spectrum of **4**

Figure S33 ^{1}H NMR spectrum of **5** at 600 MHz in CD_3OD

Figure S34 ^{13}C NMR spectrum of **5** at 150 MHz in CD_3OD

Figure S35 HSQC spectrum of **5** in CD_3OD

Figure S36 ^{1}H-^{1}H COSY spectrum of **5** in CD_3OD

Figure S37 HMBC spectrum of **5** in CD_3OD

Figure S38 HRESIMS spectrum of **5**

Figure S39 IR spectrum of **5**

Figure S40 ^{1}H NMR spectrum of **6** at 400 MHz in DMSO-d_6

Figure S41 ^{13}C NMR spectrum of **6** at 100 MHz in DMSO-d_6

Figure S42 HSQC spectrum of **6** in DMSO-d_6

Figure S43 ^{1}H-^{1}H COSY spectrum of **6** in DMSO-d_6

Figure S44 HMBC spectrum of **6** in DMSO-d_6

Figure S1. ^{1}H NMR spectrum of **1** at 400 MHz in CD_3OD.

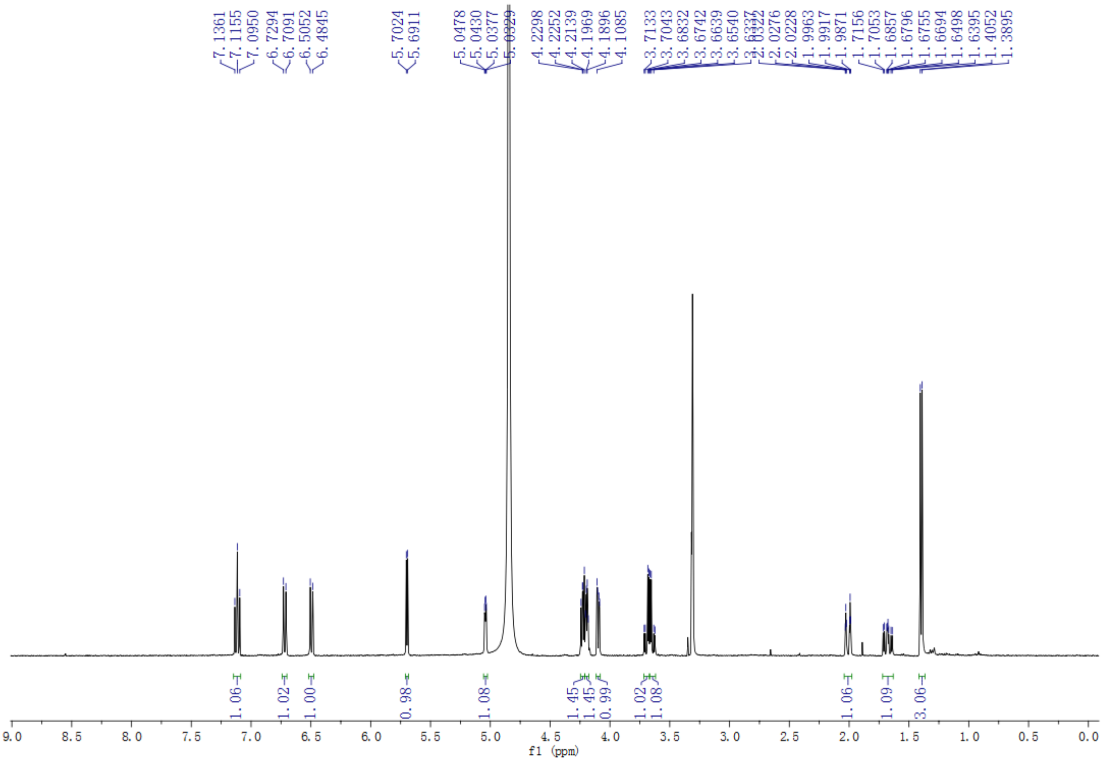

Figure S2. ^{13}C NMR spectrum of **1** at 100 MHz in CD_3OD.

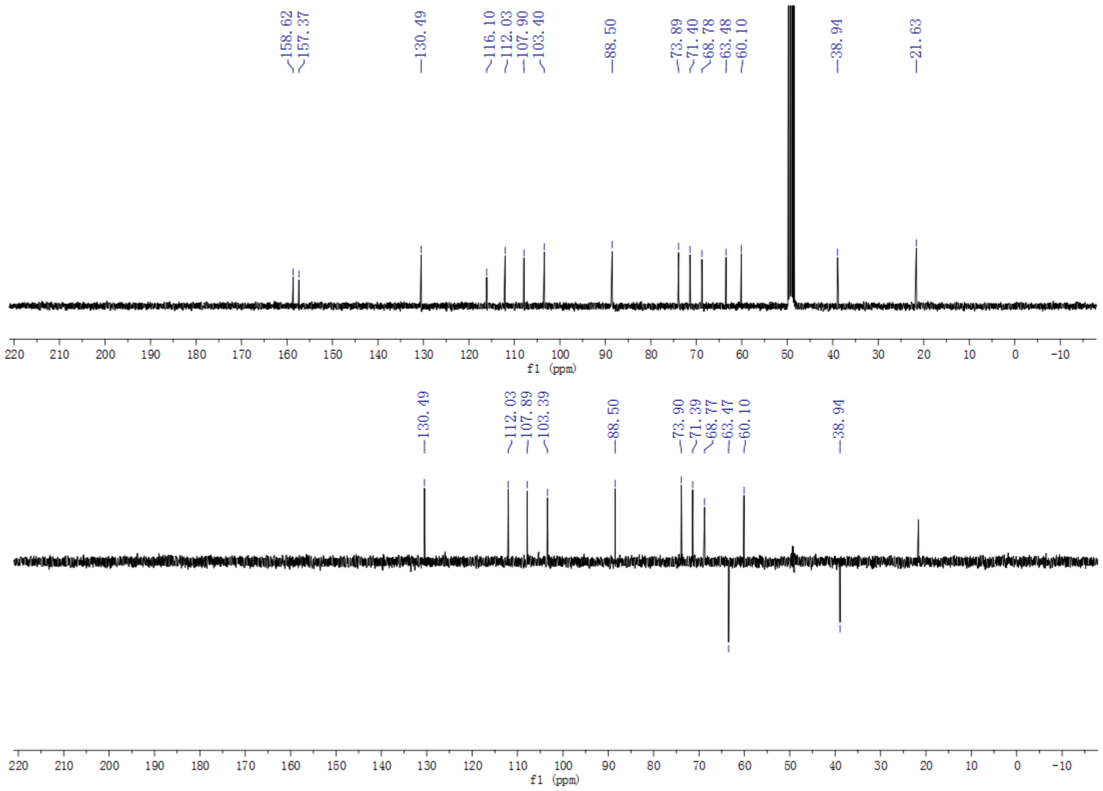

Figure S3. HSQC spectrum of **1** in CD_3OD.

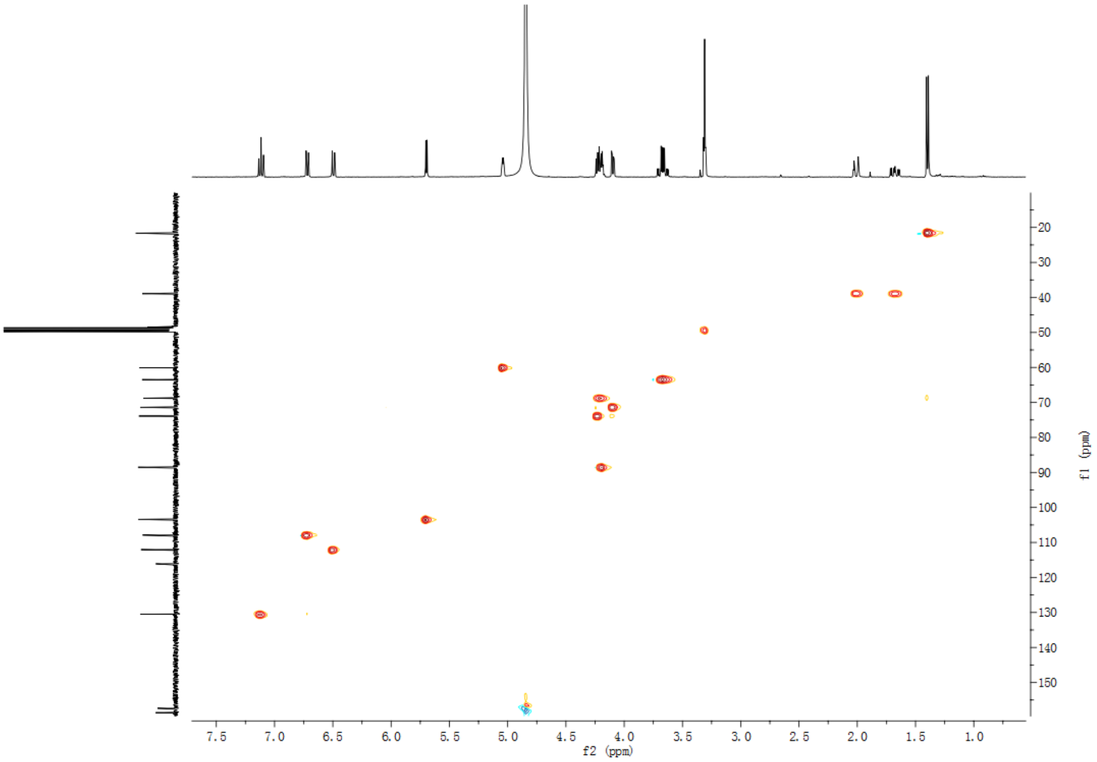

Figure S4. ^1H–^1H COSY spectrum of **1** in CD_3OD.

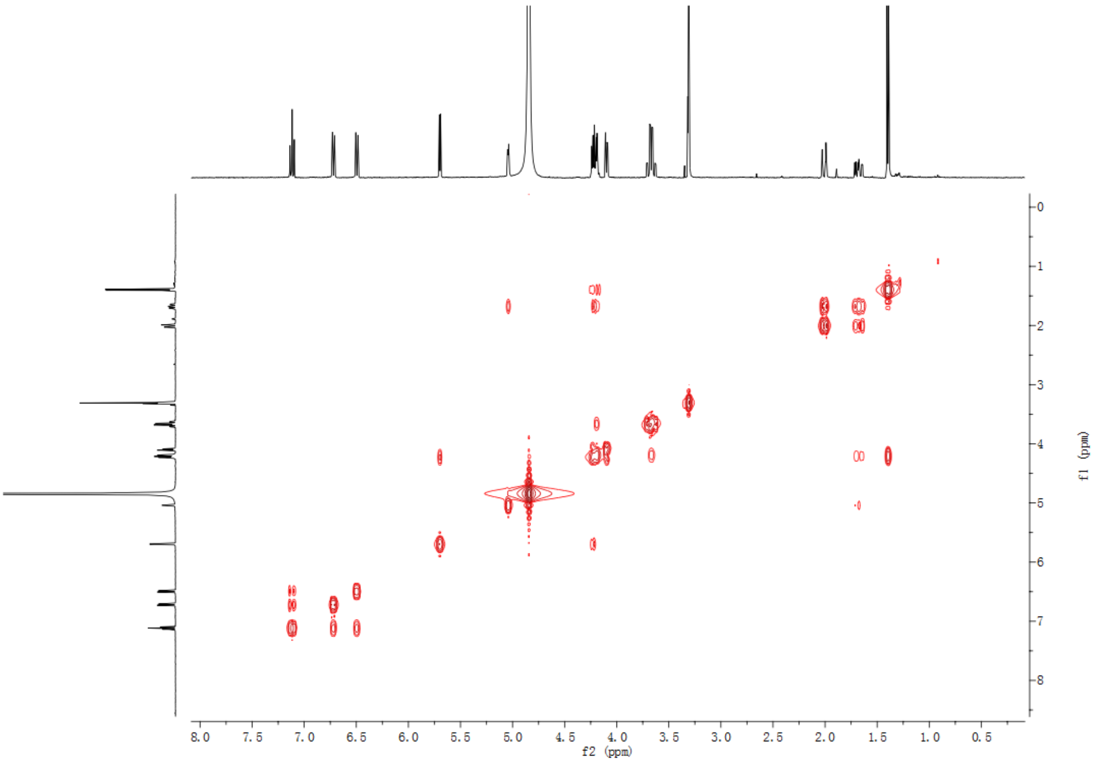

Figure S5. HMBC spectrum of **1** in CD_3OD.

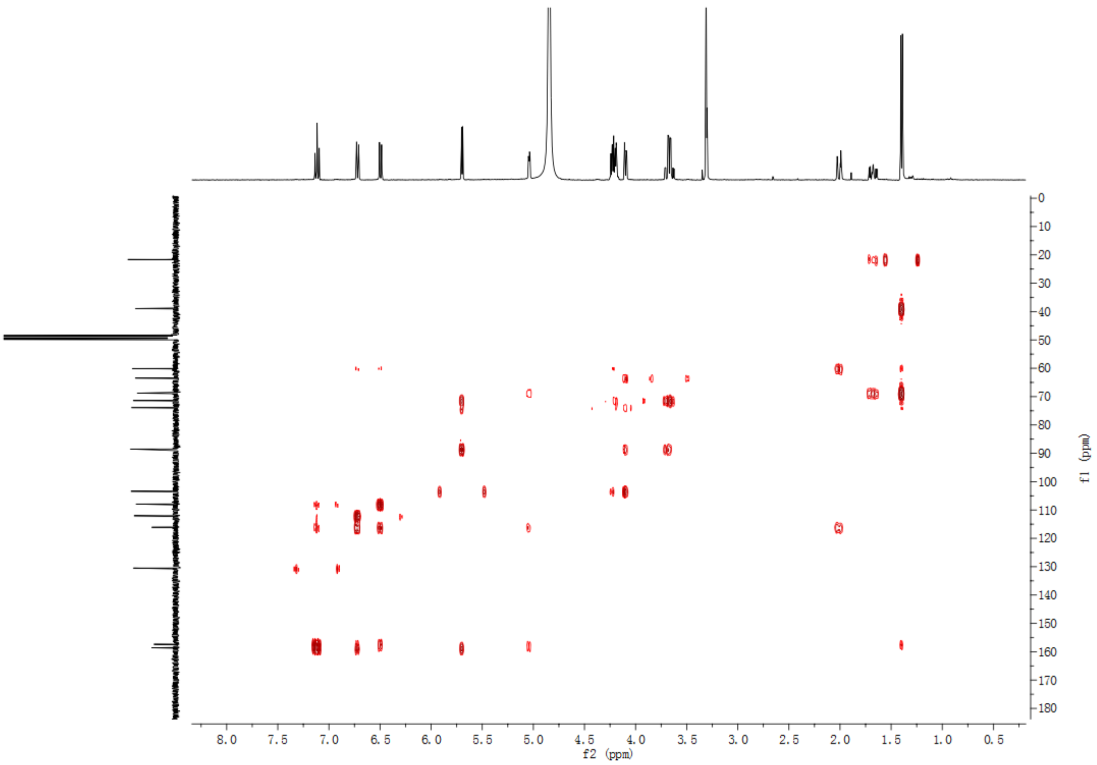

Figure S6. HRESIMS spectrum of **1**.

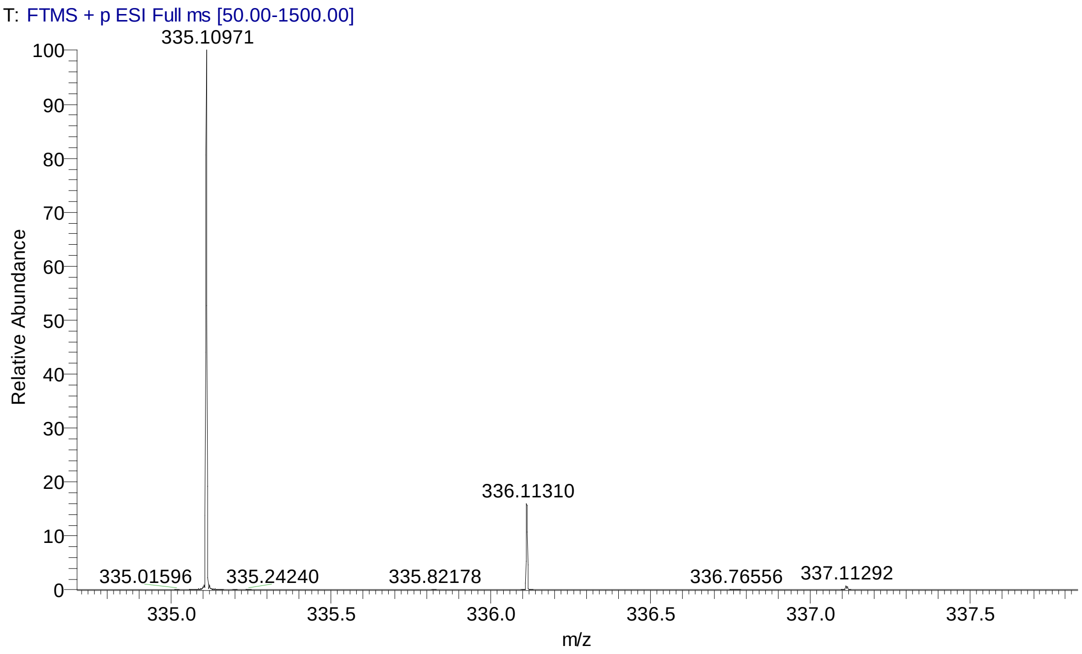

Figure S7. IR spectrum of **1**.

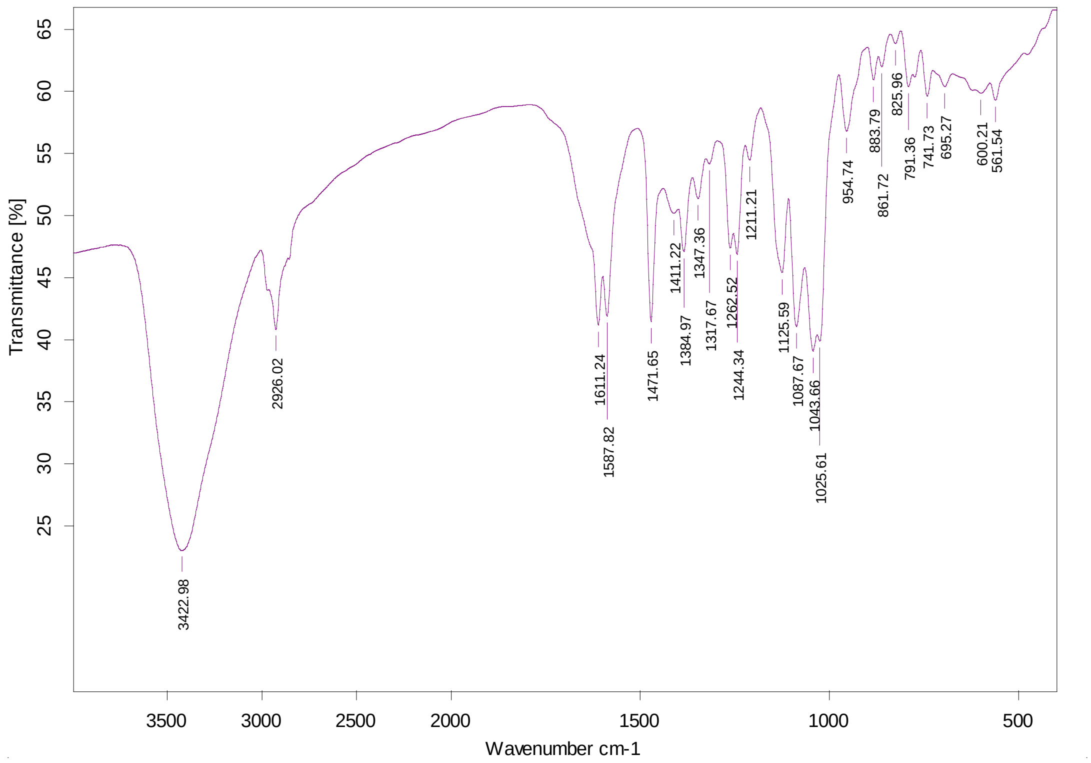

Figure S8. UV spectrum of **1**.

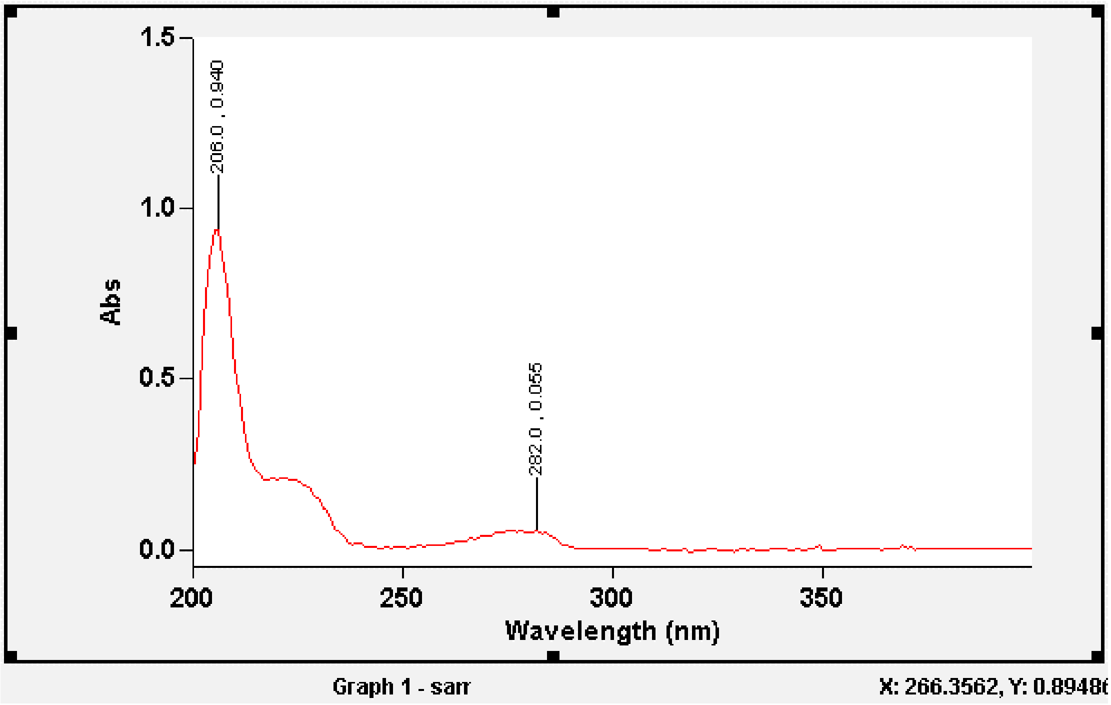

Figure S9. ¹H NMR spectrum of **2** at 400 MHz in CD3OD.

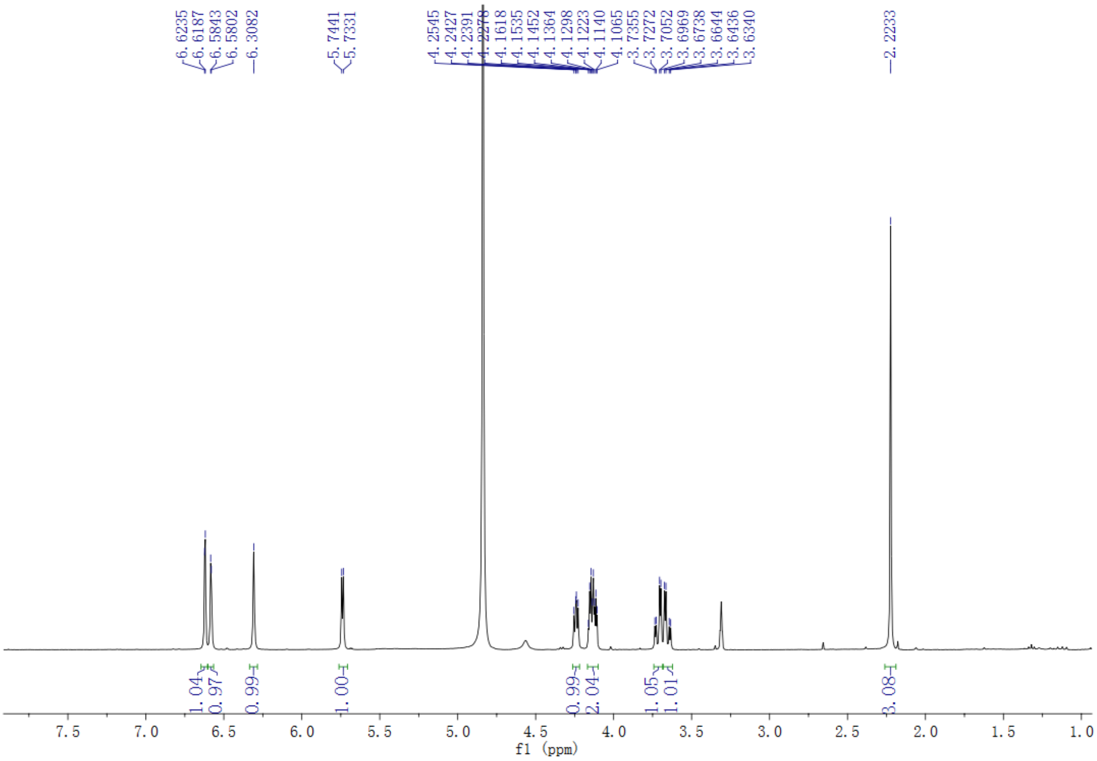

Figure S10. ¹³C NMR spectrum of **2** at 100 MHz in CD3OD.

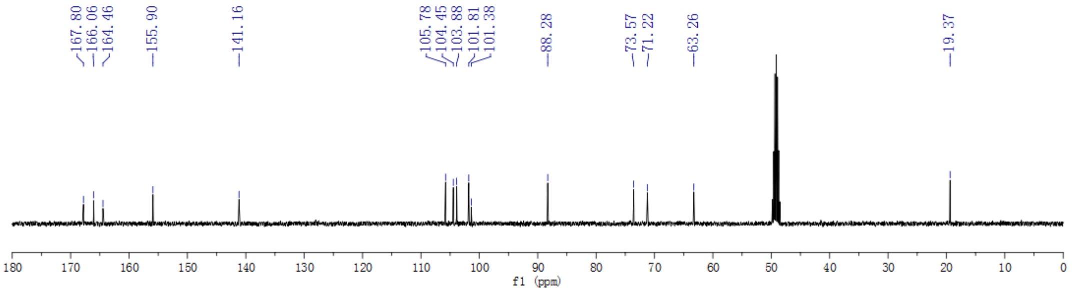

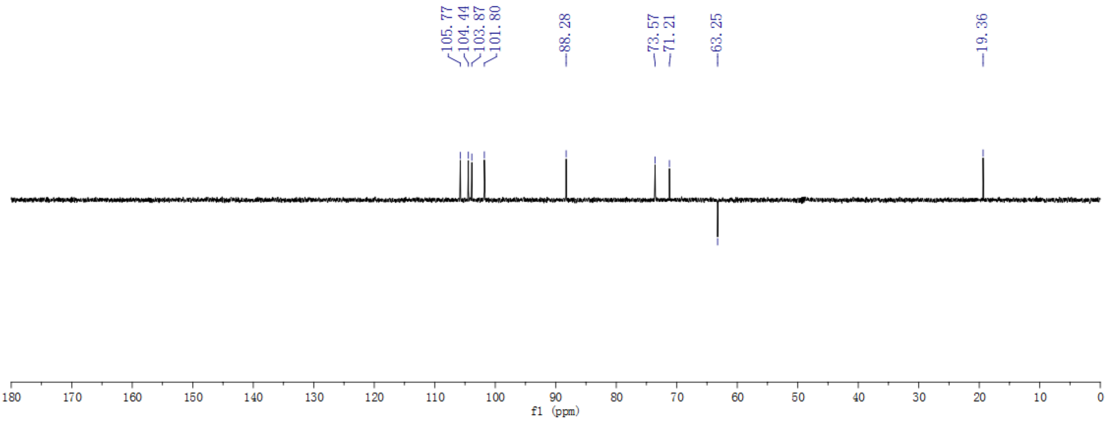

Figure S11. HSQC spectrum of **2** in CD_3OD.

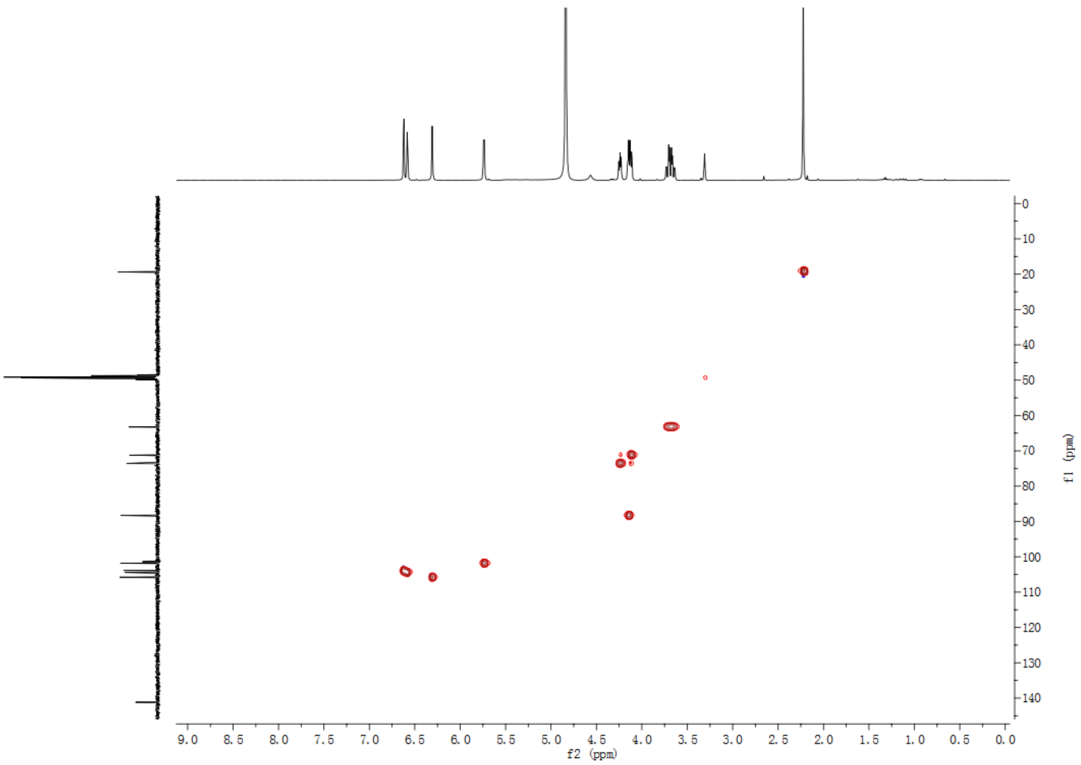

Figure S12. ^1H–^1H COSY spectrum of **2** in CD_3OD.

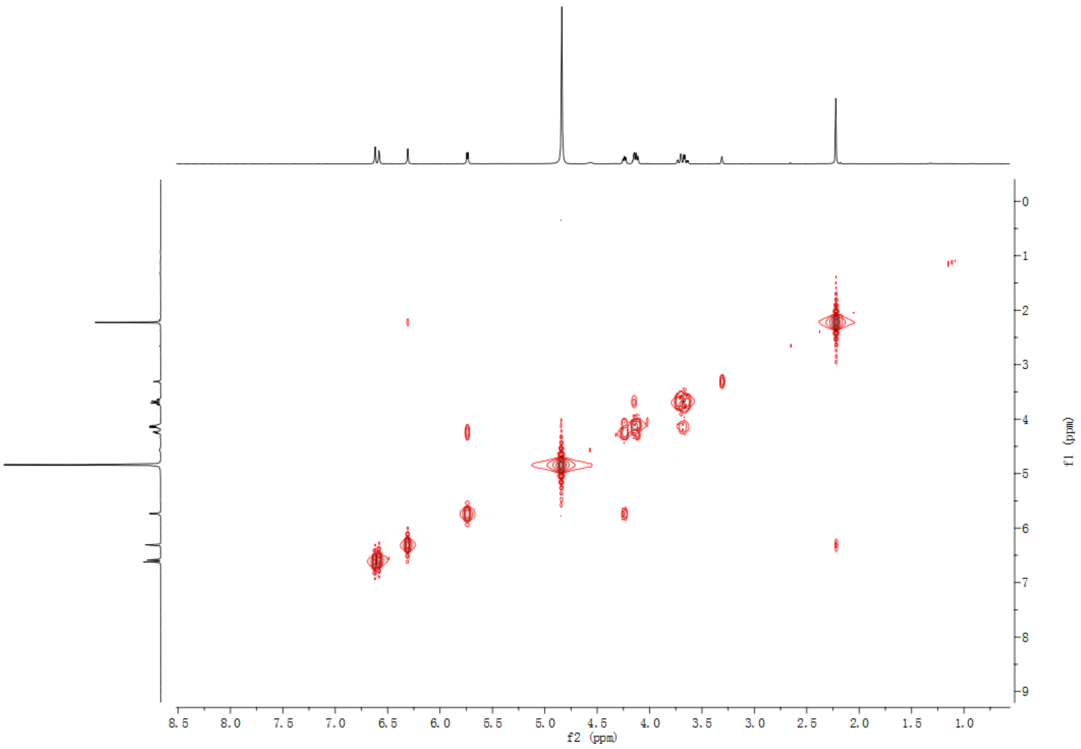

Figure S13. HMBC spectrum of **2** in CD_3OD.

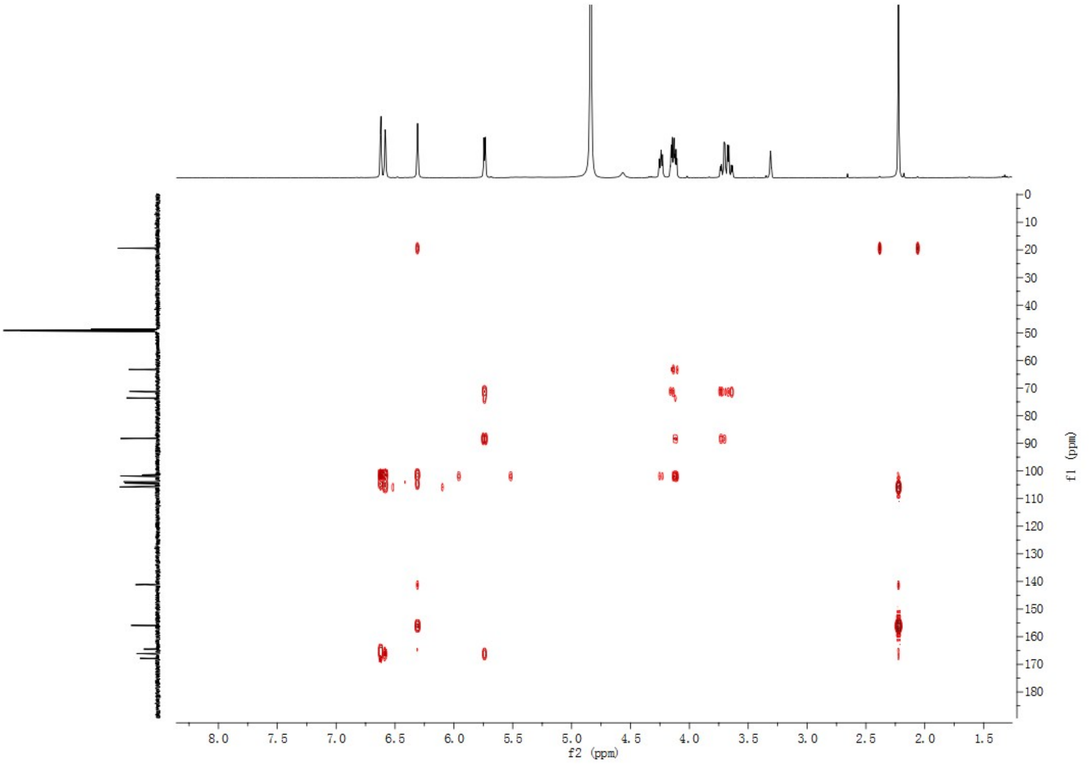

Figure S14. HRESIMS spectrum of **2**.

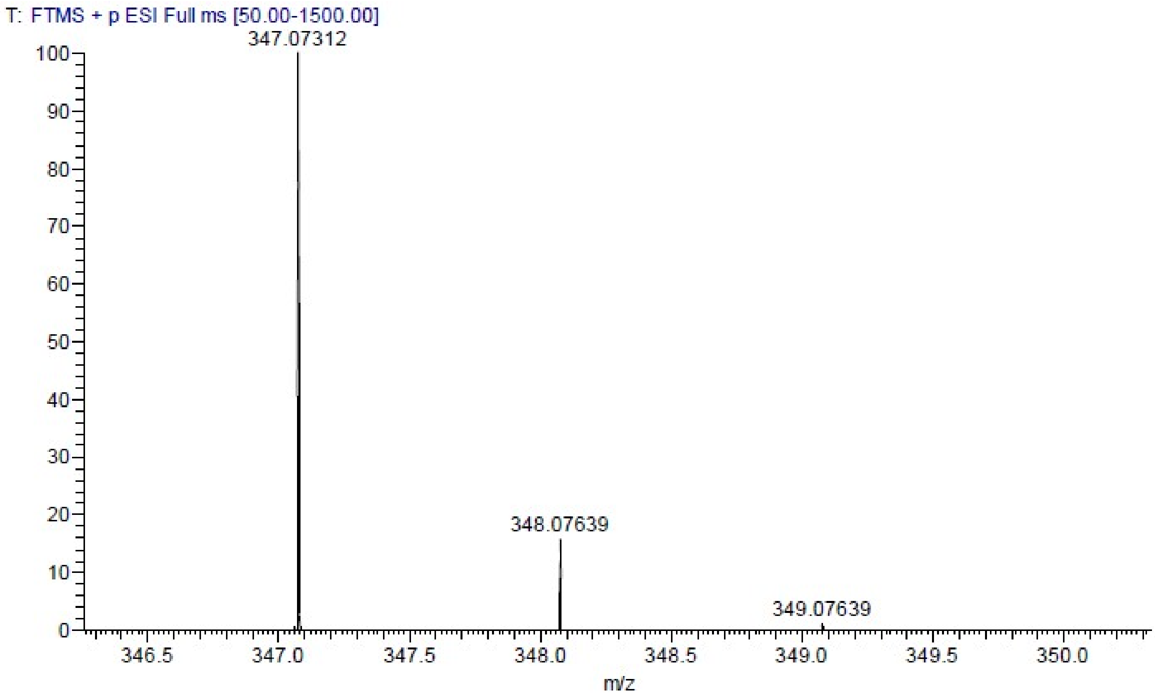

Figure S15. IR spectrum of **2**.

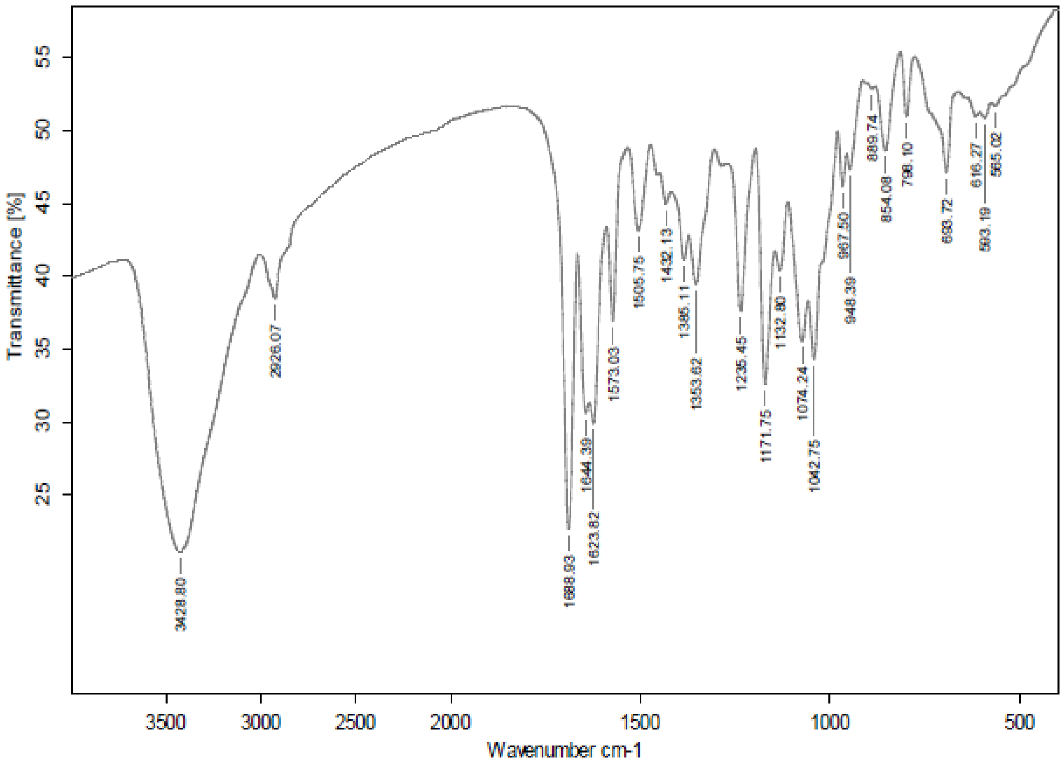

Figure S16. UV spectrum of **2**.

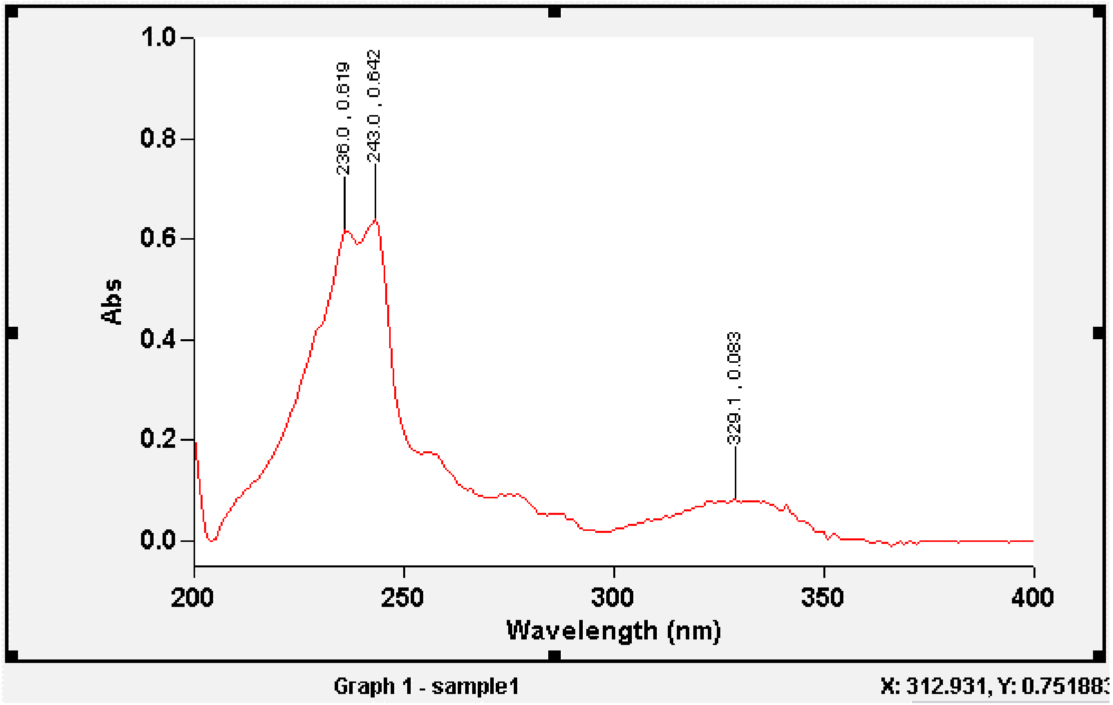

Figure S17. ^{1}H NMR spectrum of **3** at 400 MHz in C_5D_5N.

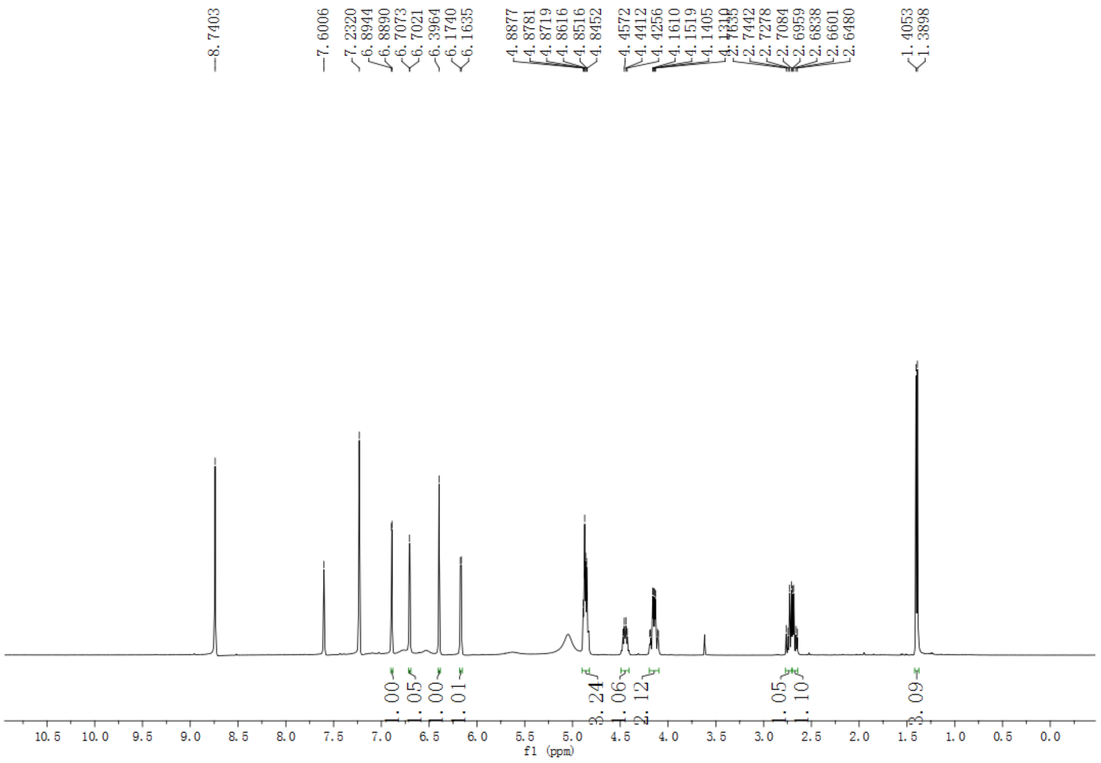

Figure S18. ^{13}C NMR spectrum of **3** at 100 MHz in C_5D_5N.

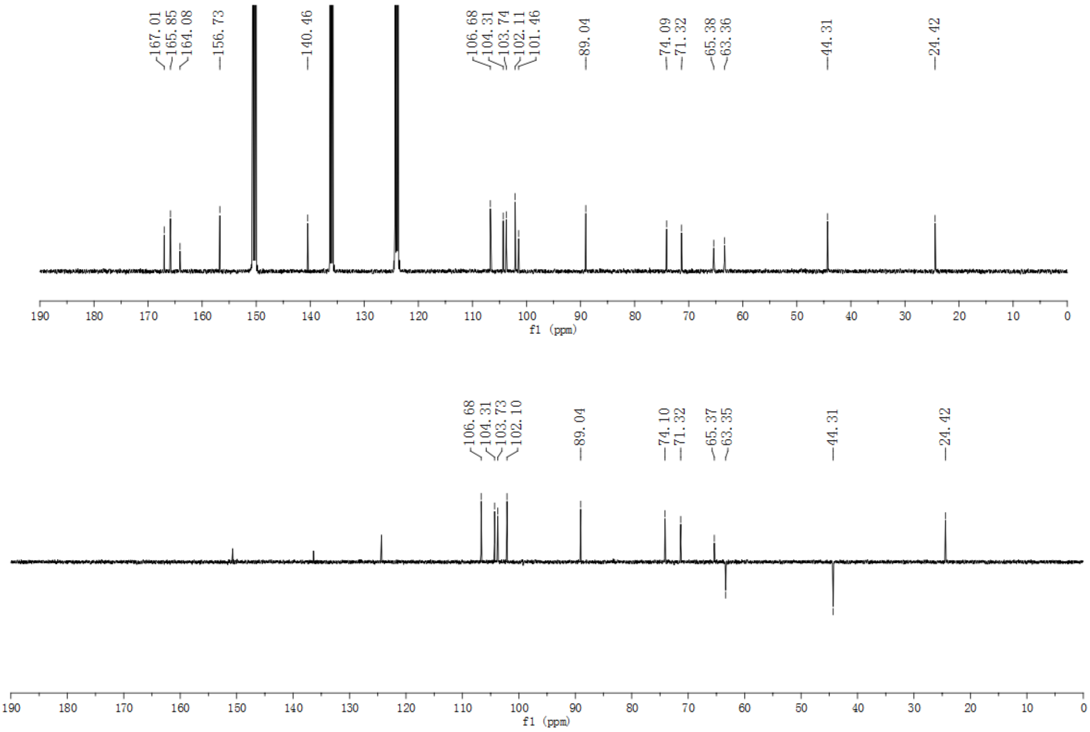

Figure S19. HSQC spectrum of **3** in C_5D_5N.

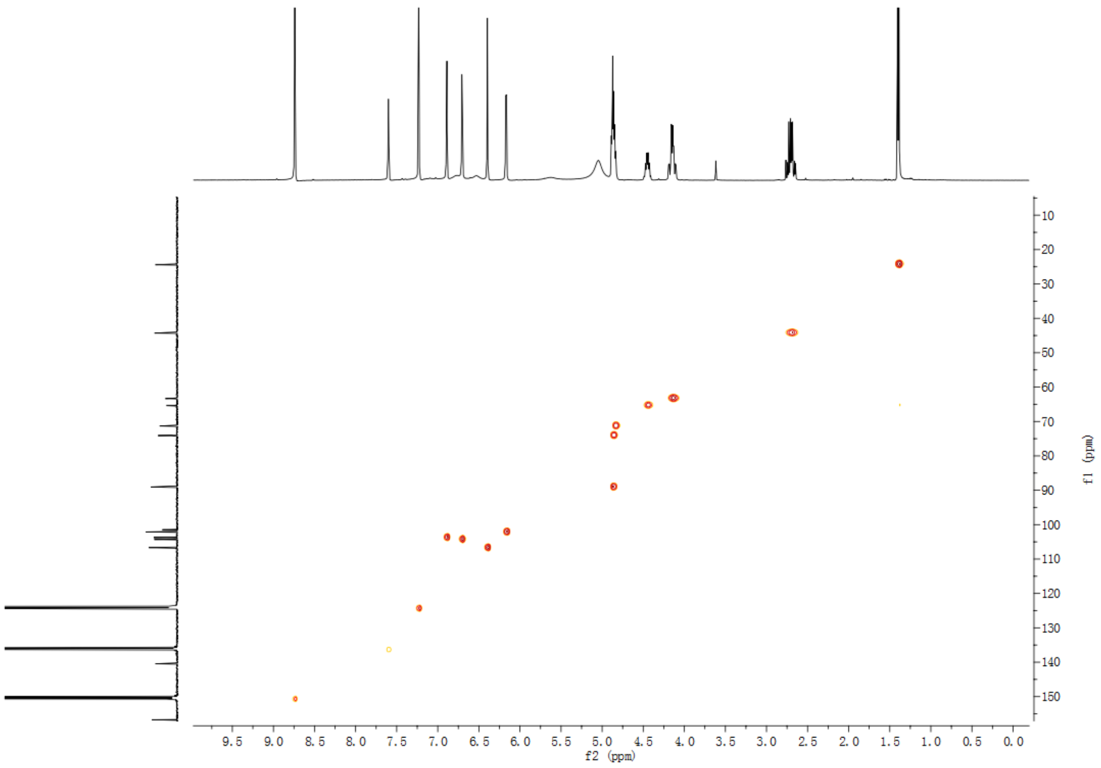

Figure S20. ^{1}H–^{1}H COSY spectrum of **3** in C_5D_5N.

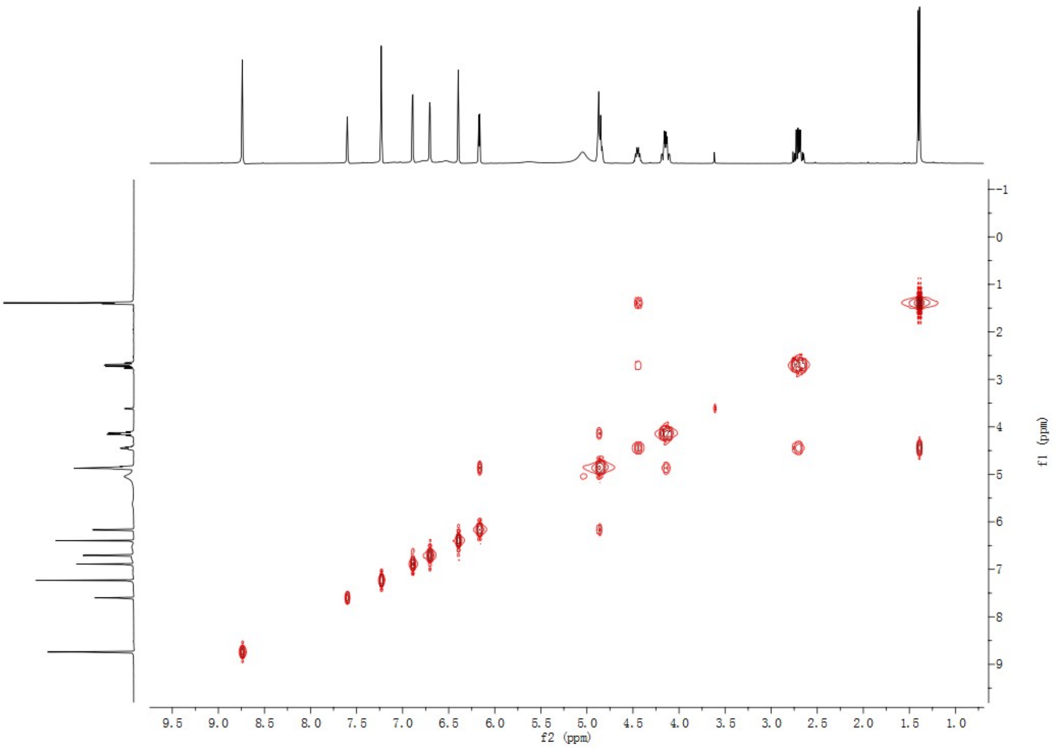

Figure S21. HMBC spectrum of **3** in C_5D_5N.

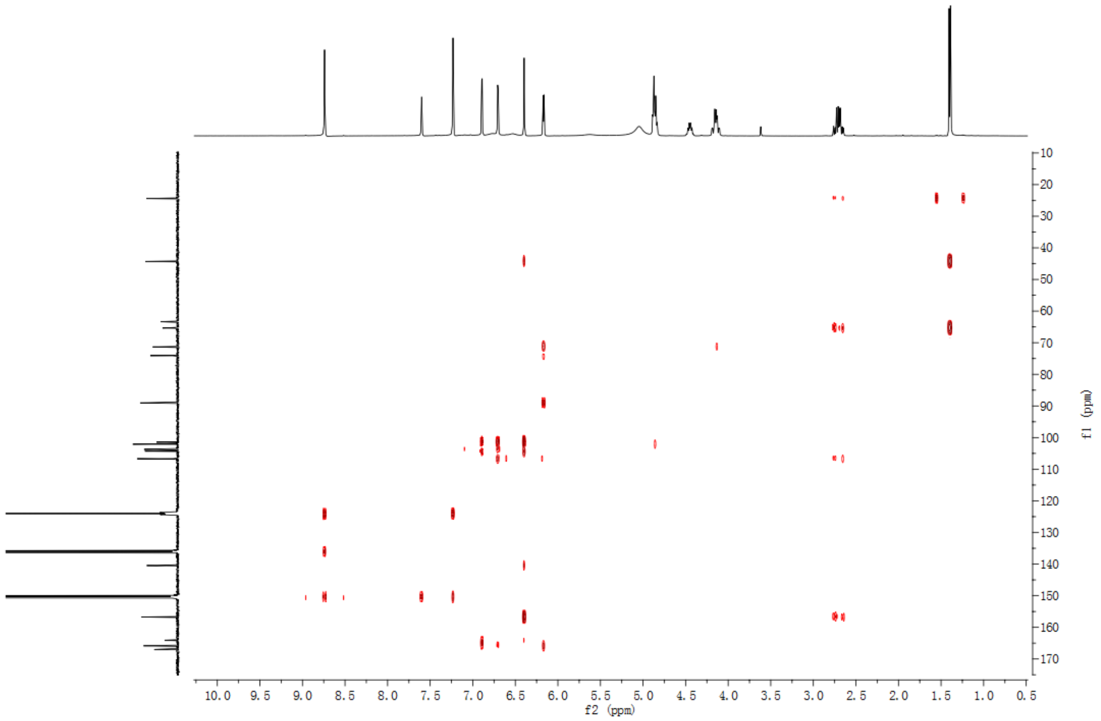

Figure S22. HRESIMS spectrum of **3**.

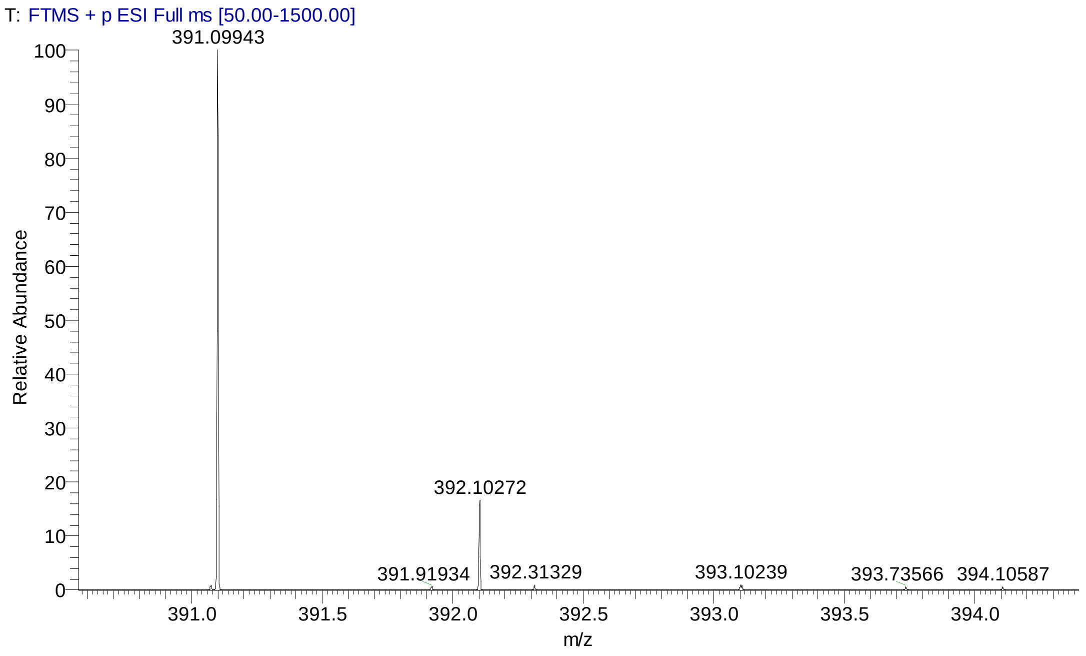

Figure S23. IR spectrum of **3**.

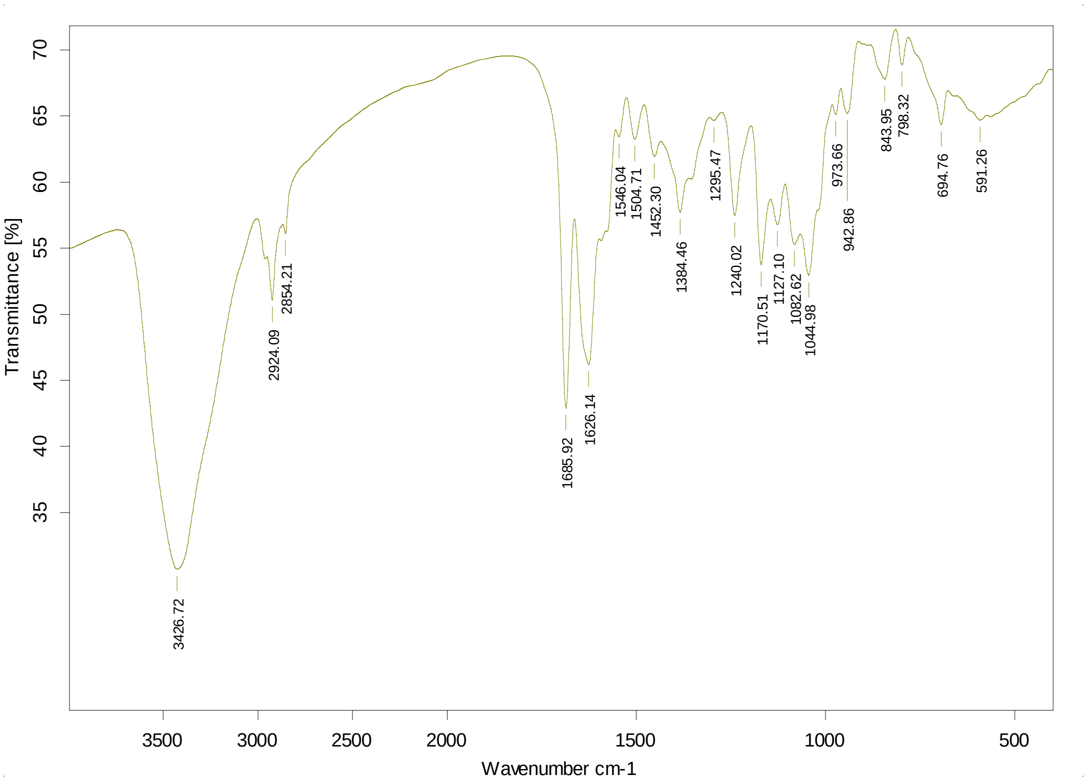

Figure S24. UV spectrum of **3**.

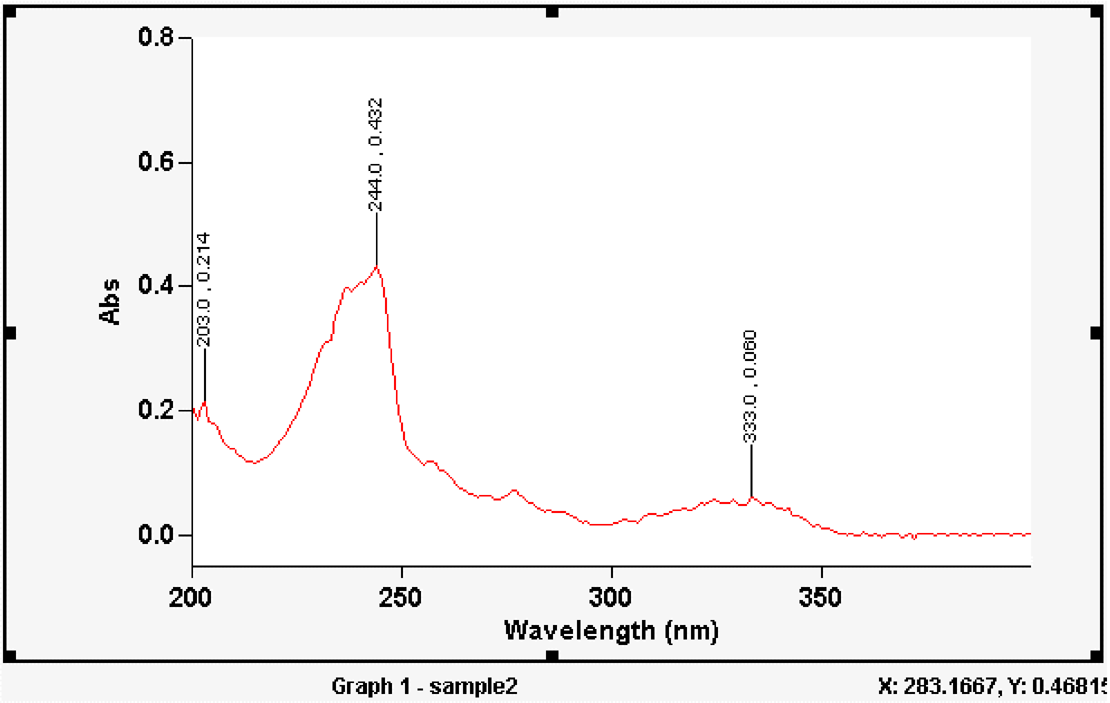

Figure S25. ^{1}H NMR spectrum of **4** at 400 MHz in DMSO-d_6.

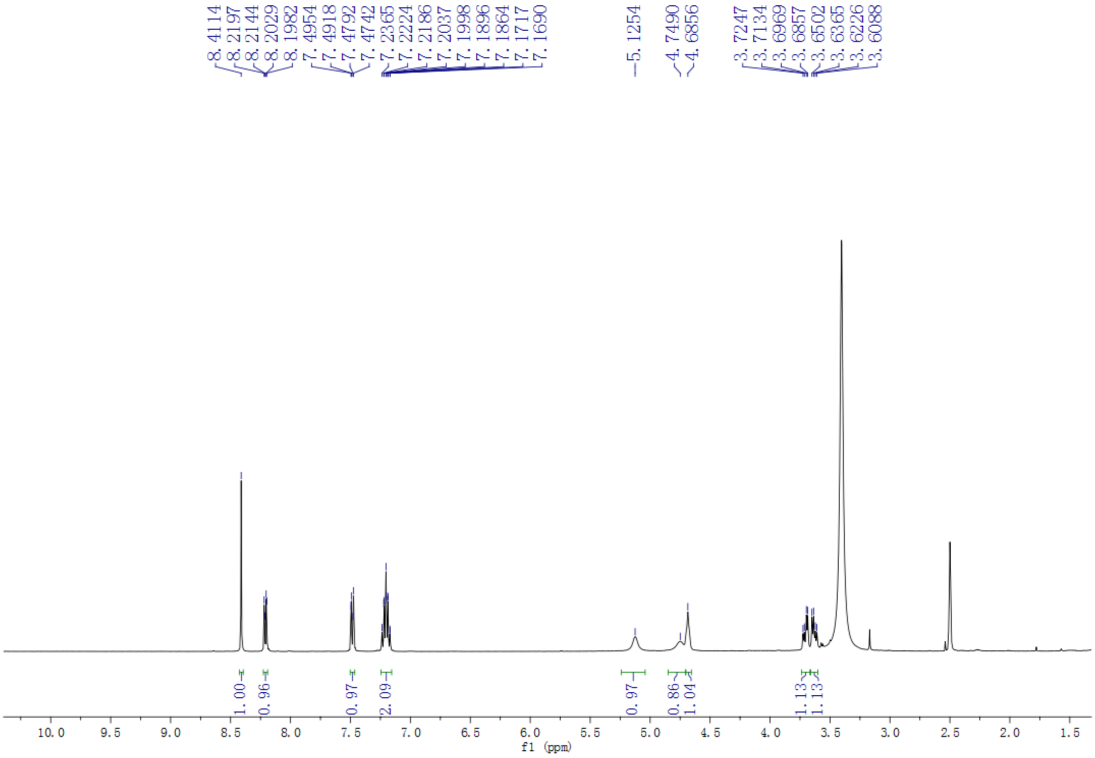

Figure S26. ^{13}C NMR spectrum of **4** at 100 MHz in DMSO-d_6.

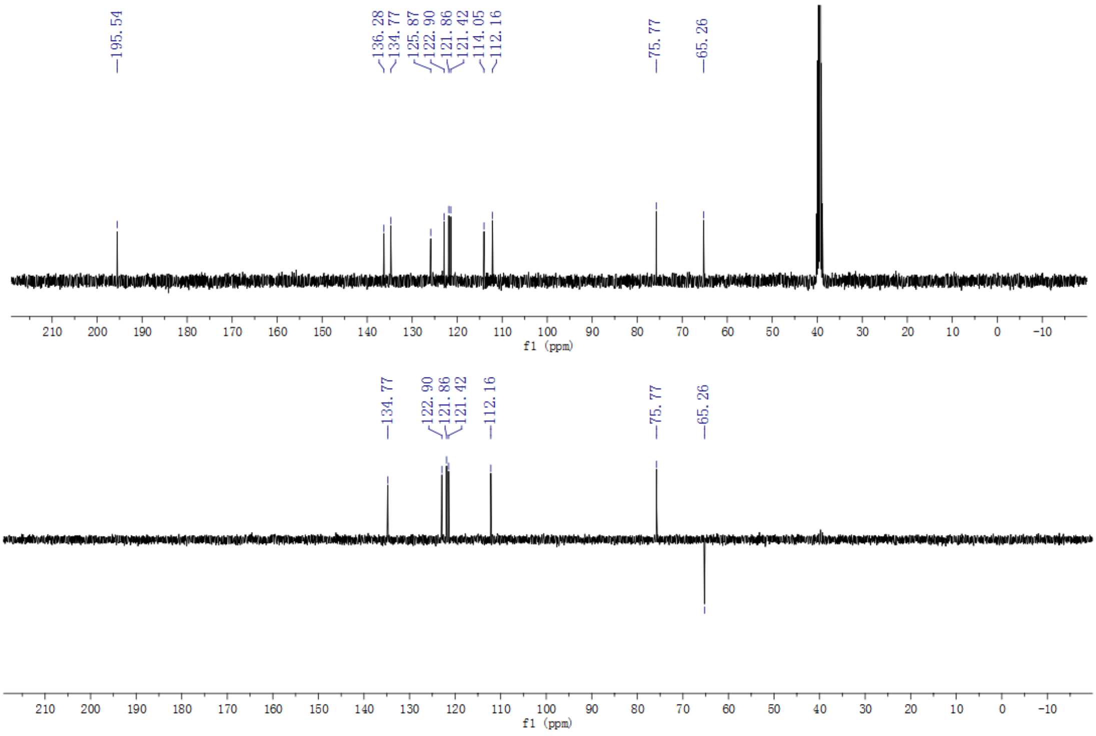

Figure S27. HSQC spectrum of **4** in DMSO-*d*6.

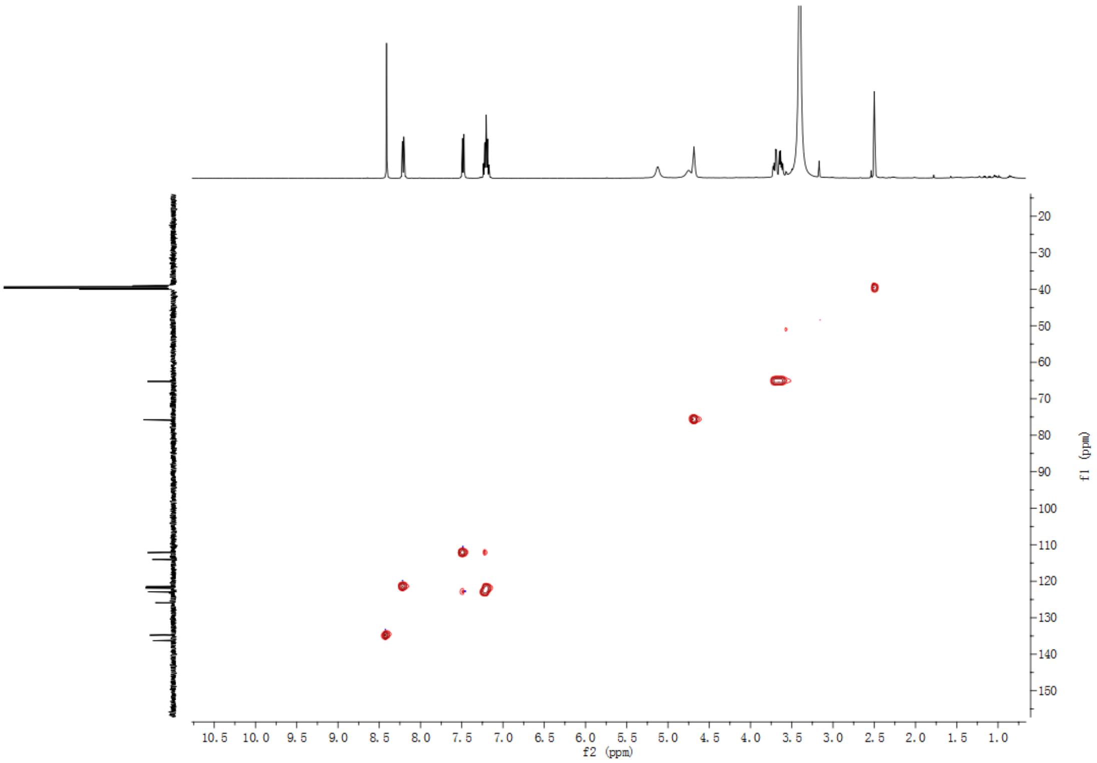

Figure S28. ^{1}H–^{1}H COSY spectrum of **4** in DMSO-*d*6.

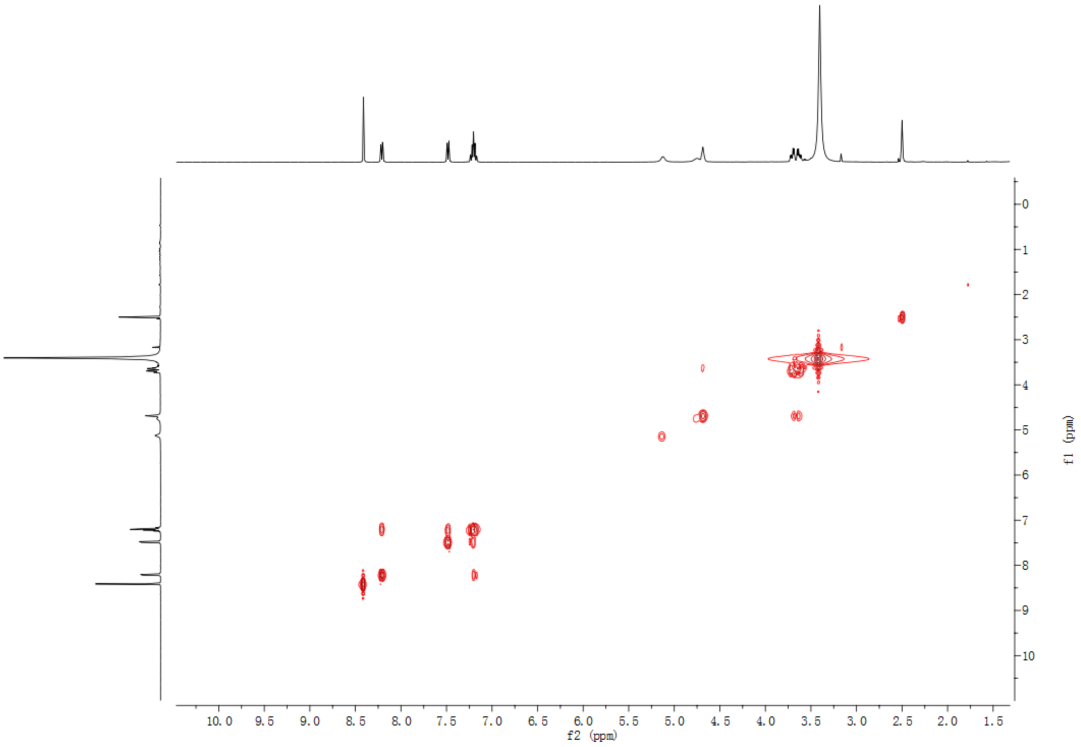

Figure S29. HMBC spectrum of **4** in DMSO-d_6.

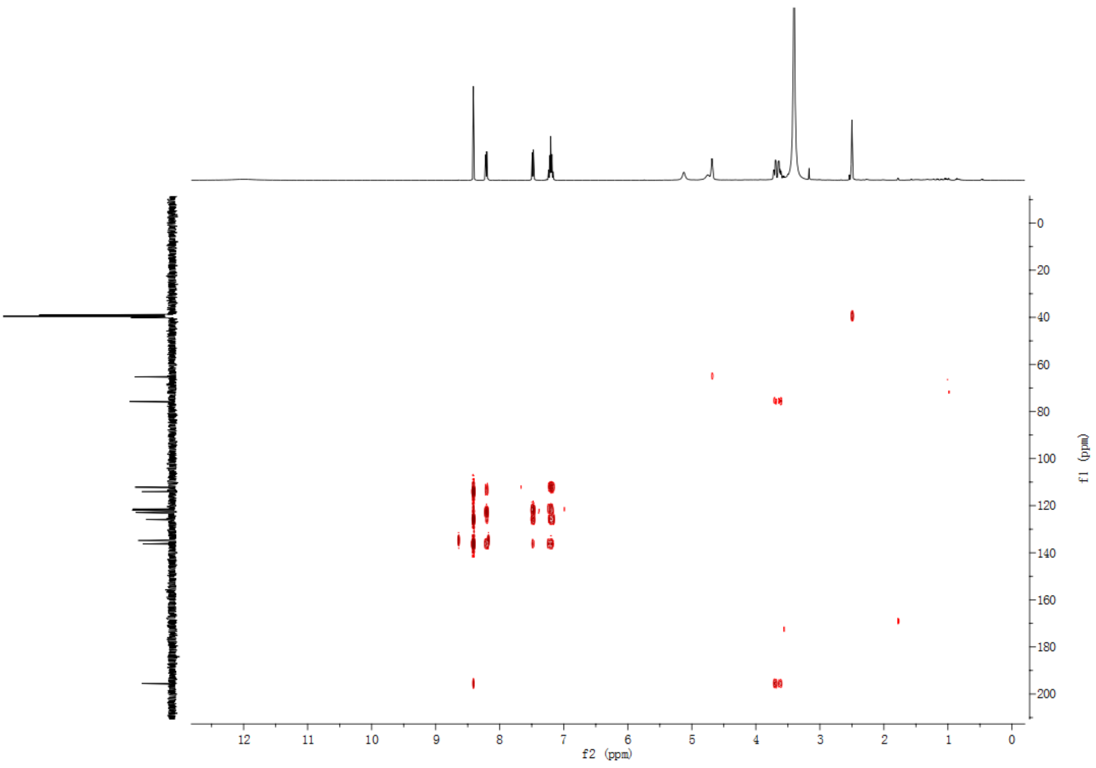

Figure S30. HRESIMS spectrum of **4**.

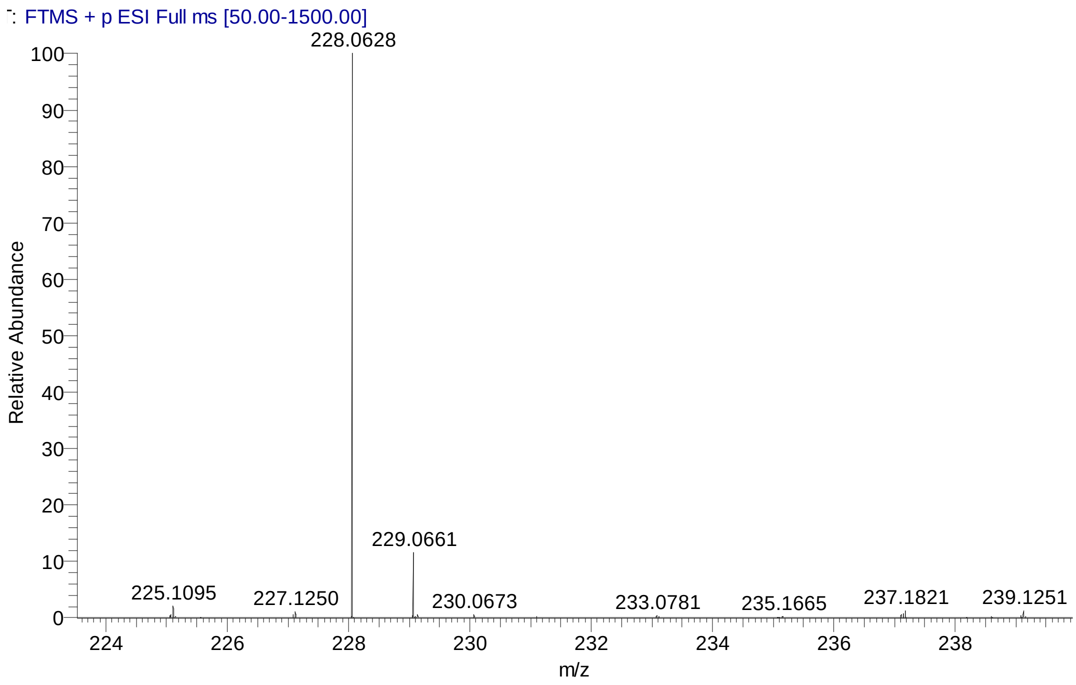

Figure S31. IR spectrum of **4**.

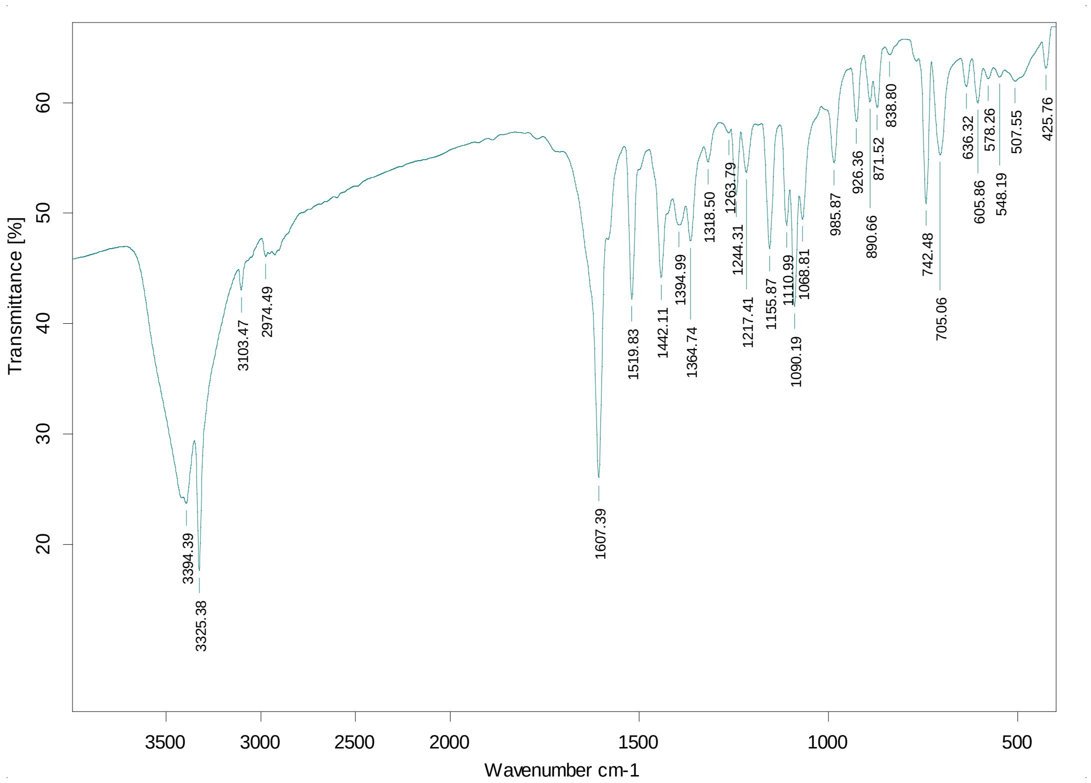

Figure S32. UV spectrum of **4**.

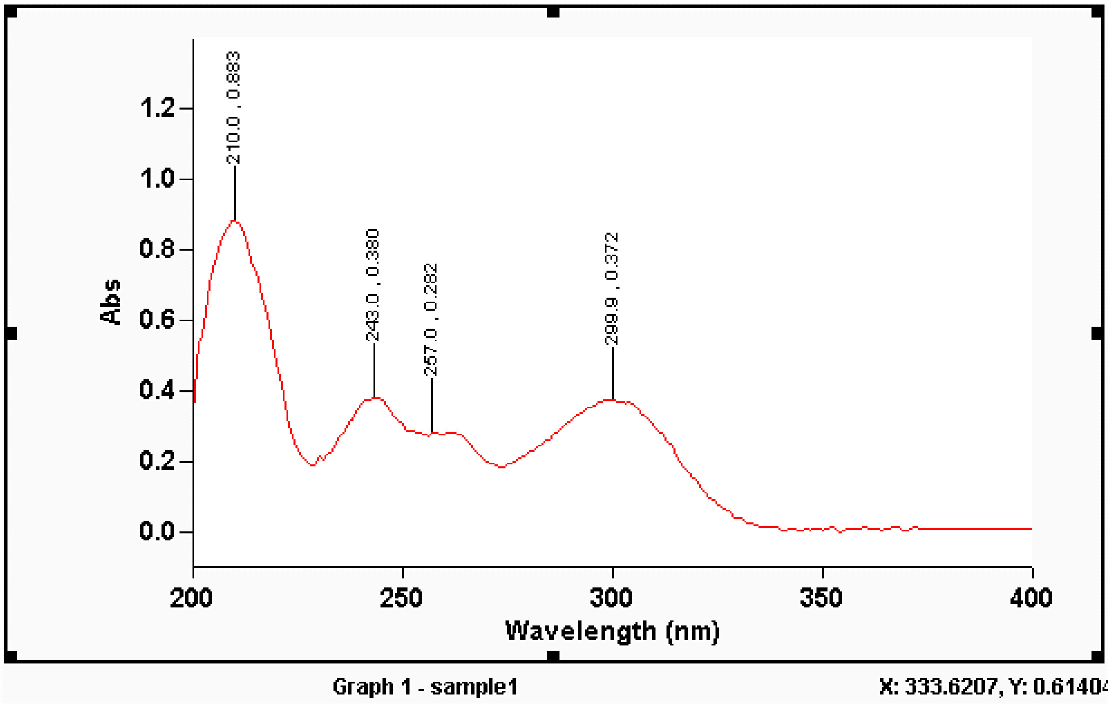

Figure S33. ^{1}H NMR spectrum of **5** at 600 MHz in CD_3OD.

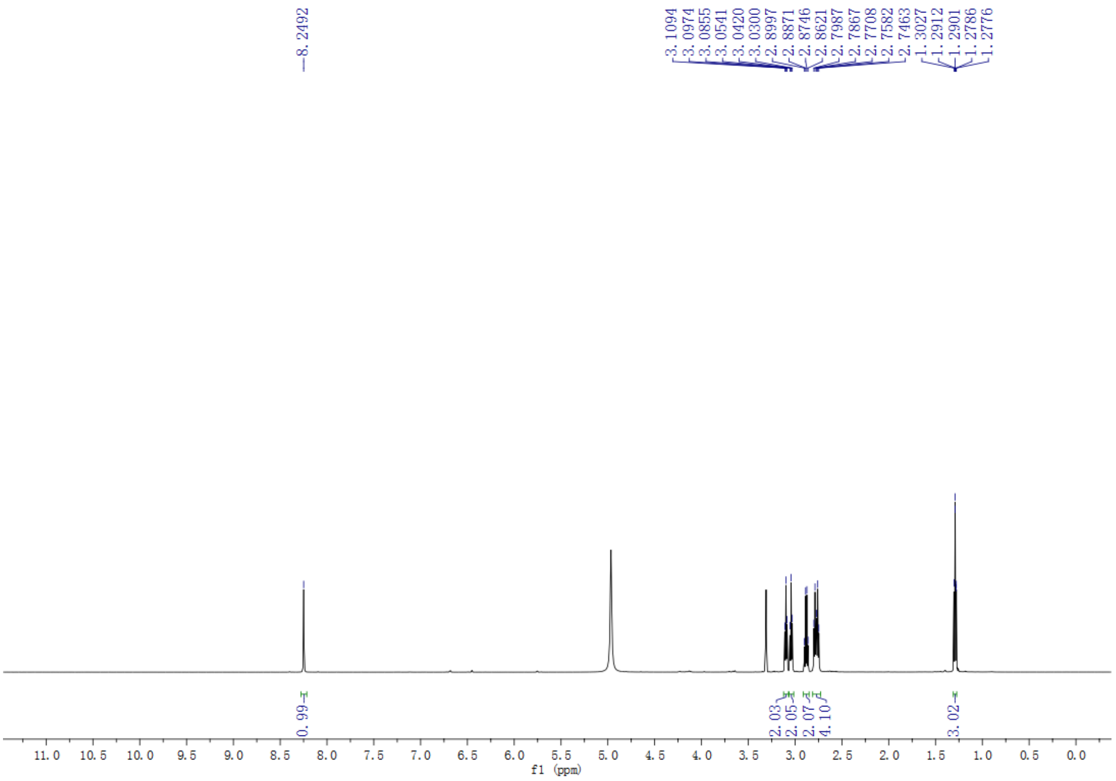

Figure S34. ^{13}C NMR spectrum of **5** at 150 MHz in CD_3OD.

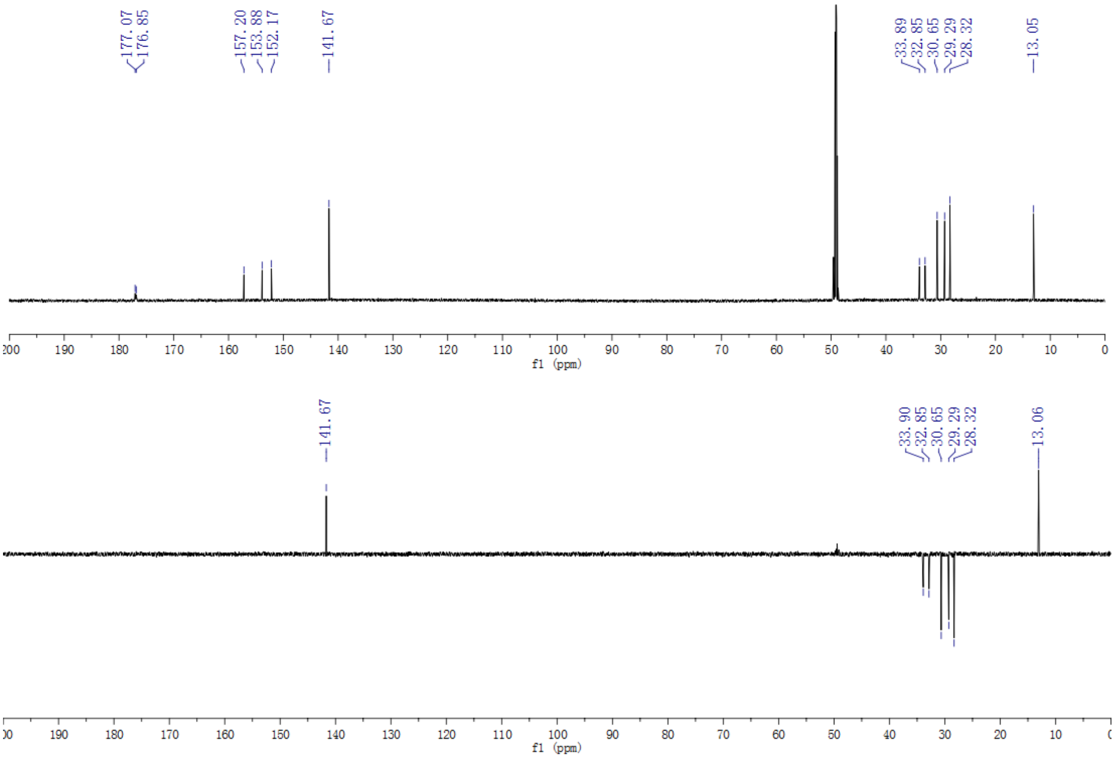

Figure S35. HSQC spectrum of **5** in CD_3OD.

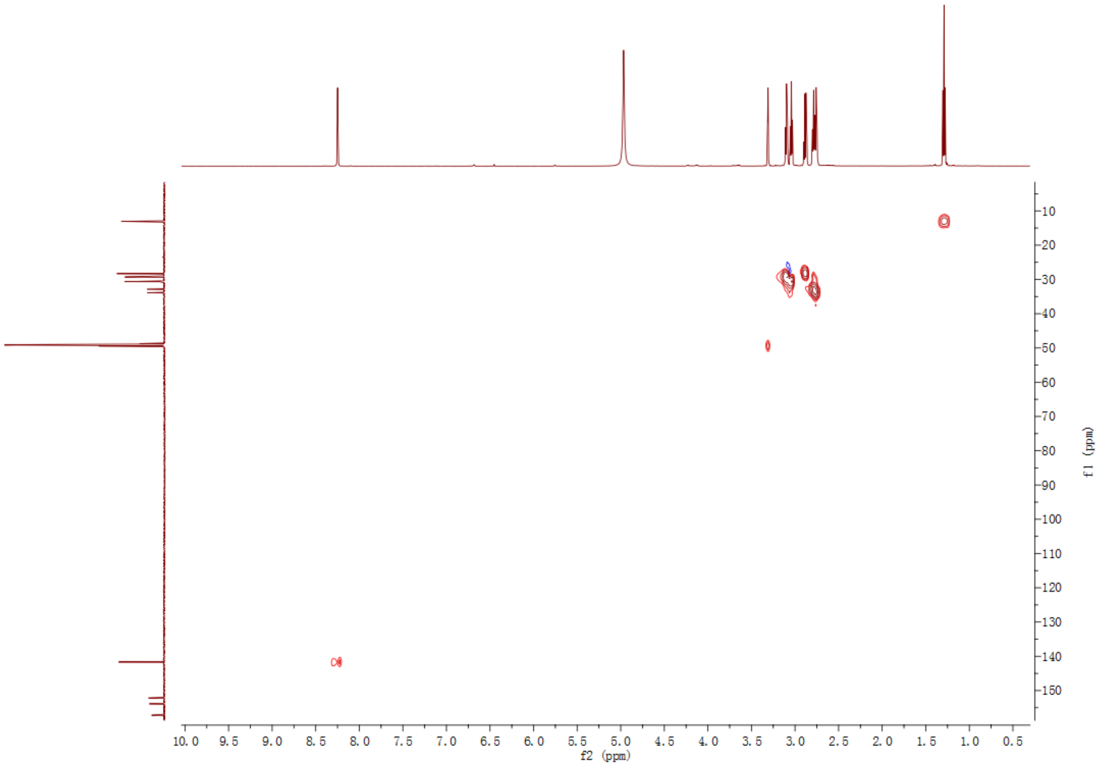

Figure S36. ¹H–¹H COSY spectrum of **5** in CD_3OD.

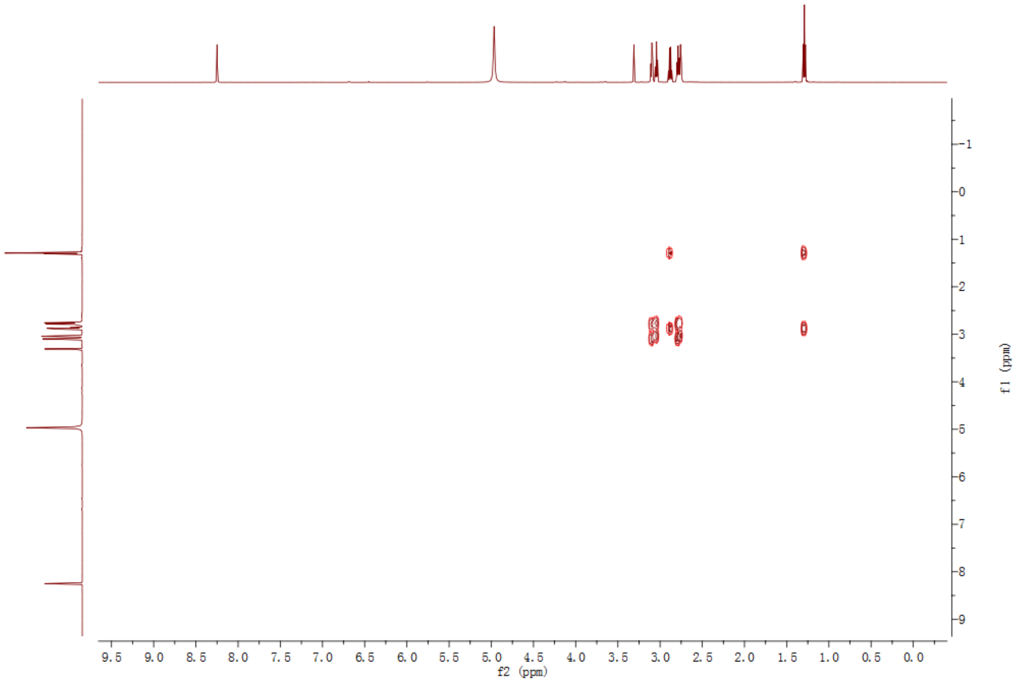

Figure S37. HMBC spectrum of **5** in CD_3OD.

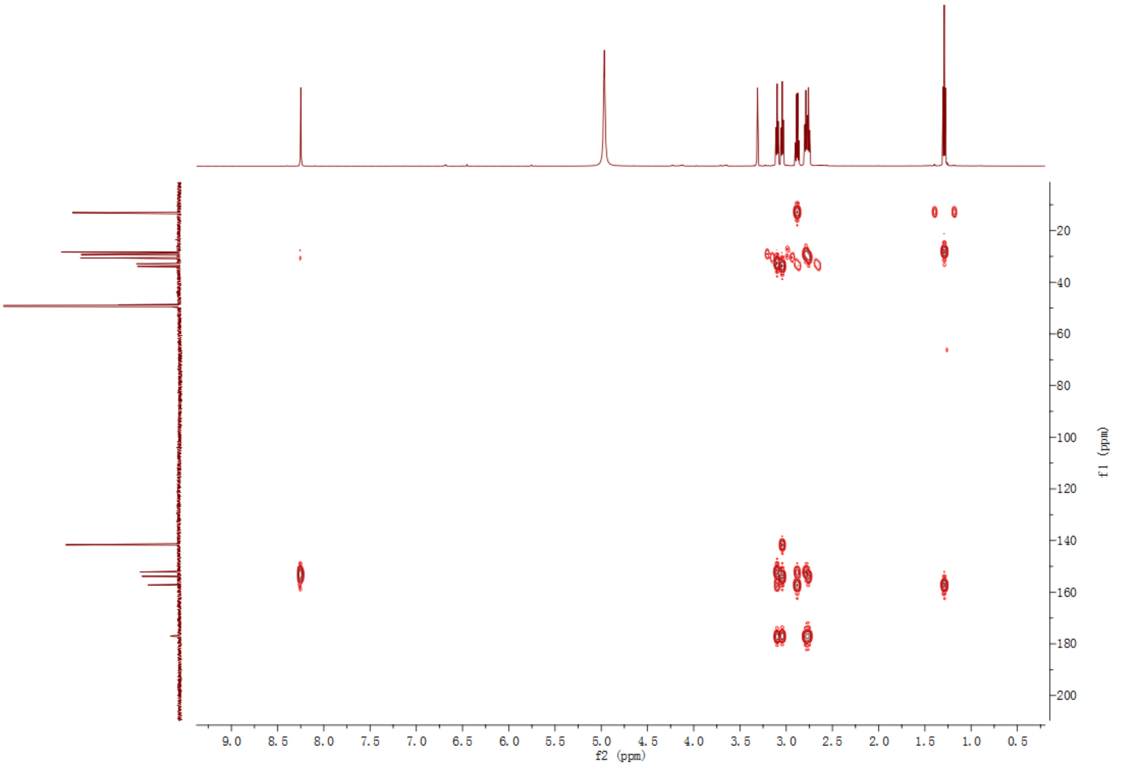

Figure S38. HRESIMS spectrum of **5**.

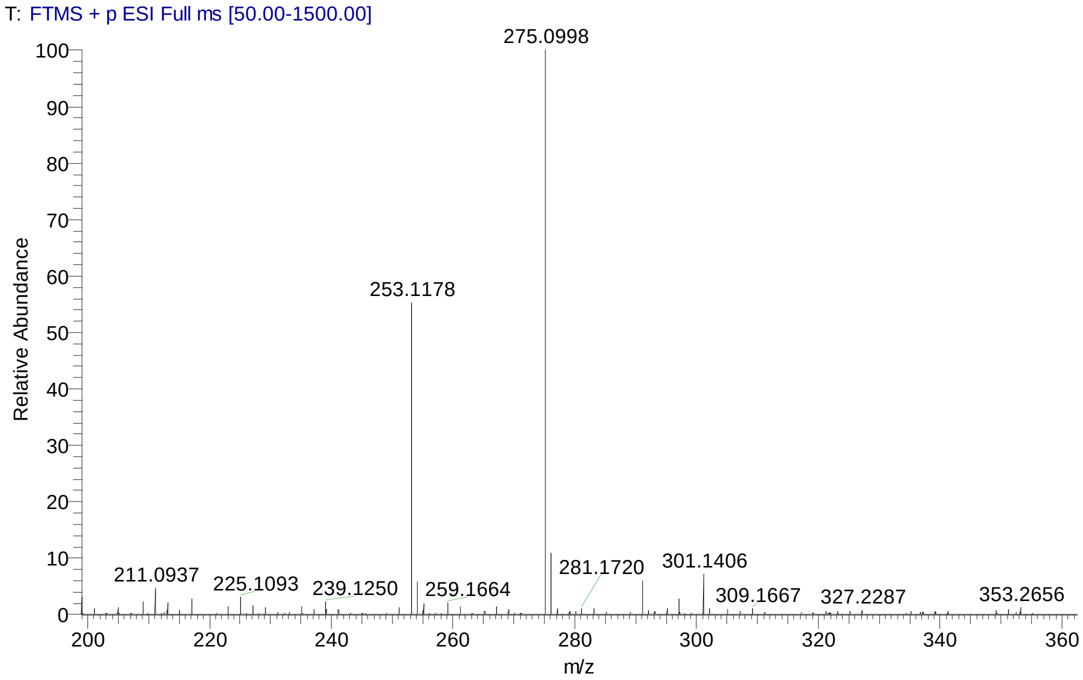

Figure S39. IR spectrum of **5**.

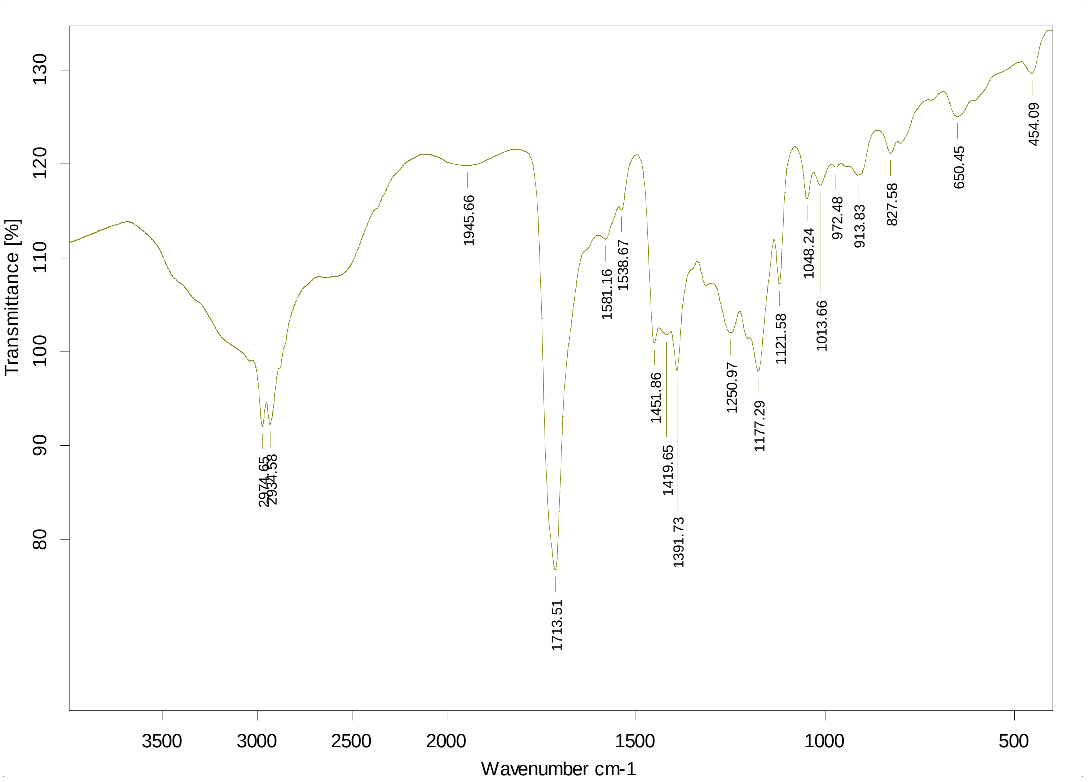

Figure S40. ^{1}H NMR spectrum of **6** at 400 MHz in DMSO-d_6.

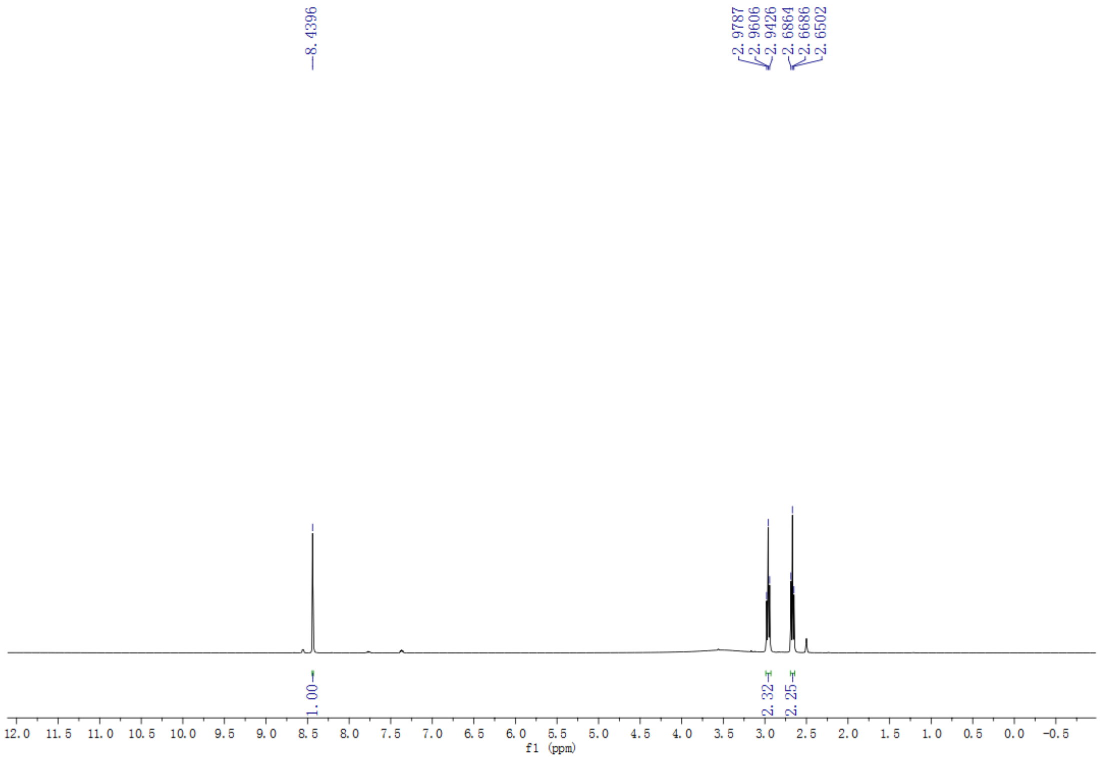

Figure S41. ^{13}C NMR spectrum of **6** at 100 MHz in DMSO-d_6.

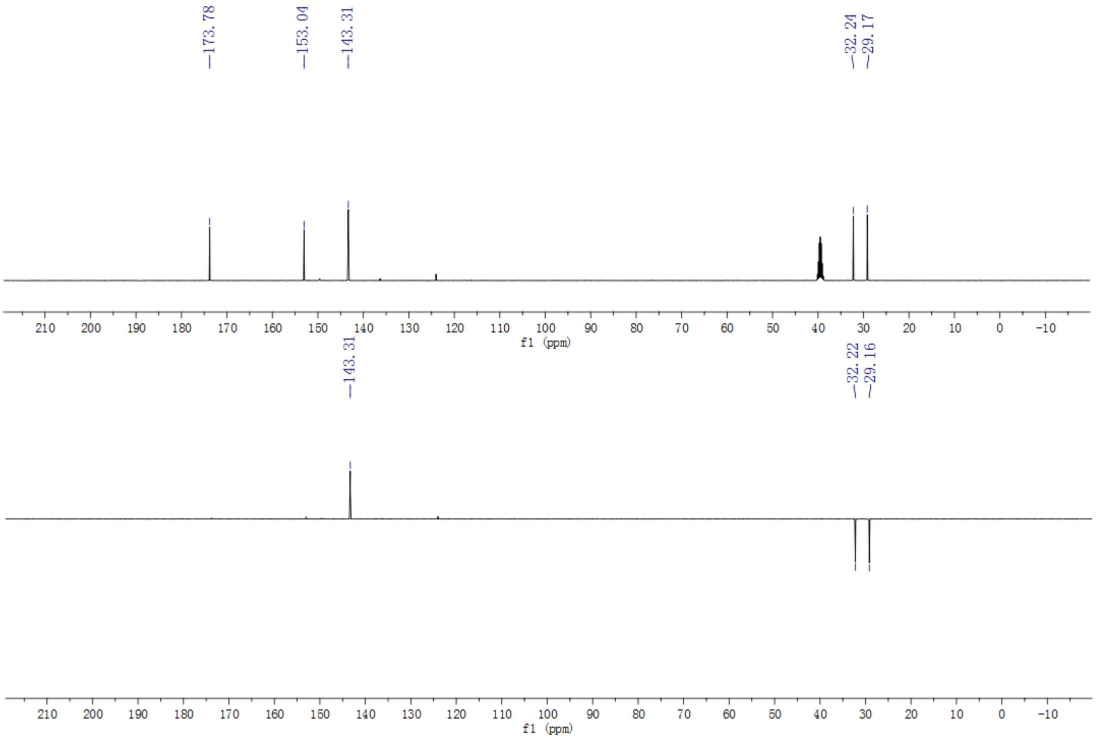

Figure S42. HSQC spectrum of **6** in DMSO-d_6.

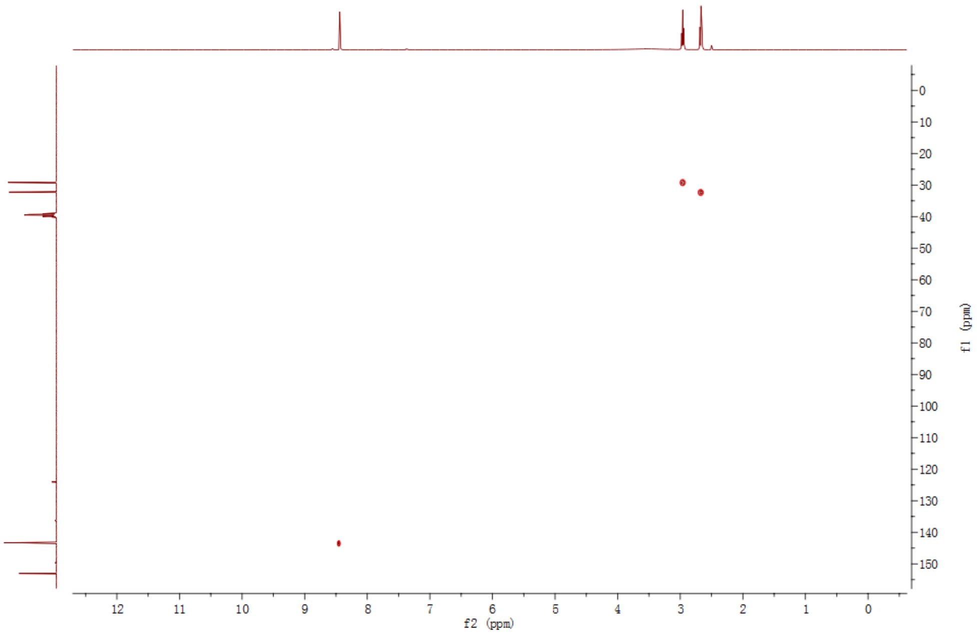

Figure S43. $^1H–^1H$ COSY spectrum of **6** in DMSO-*d*6.

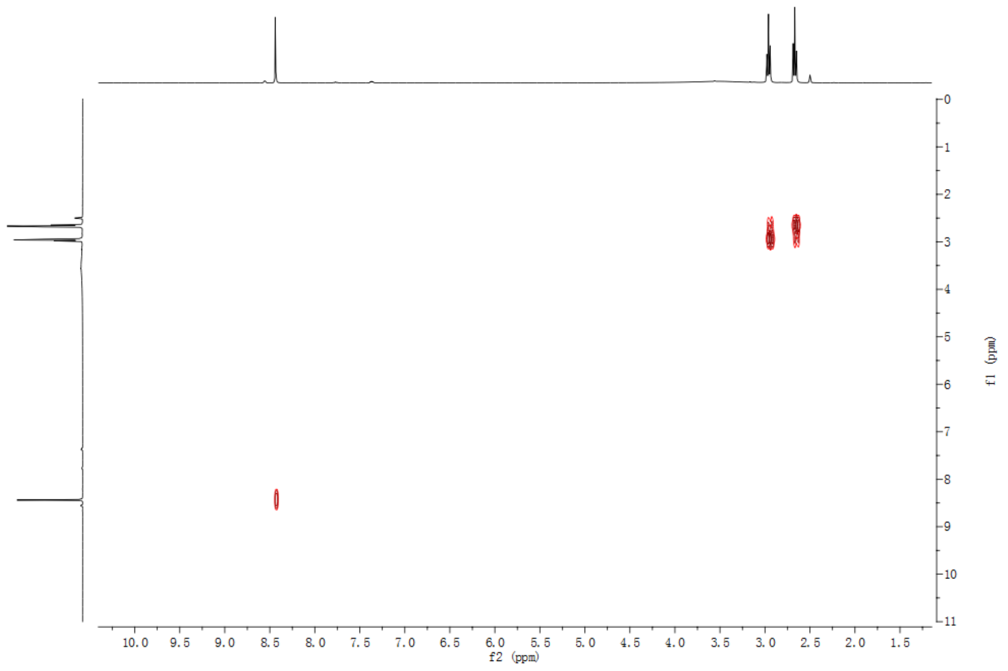

Figure S44. HMBC spectrum of **6** in DMSO-*d*6.

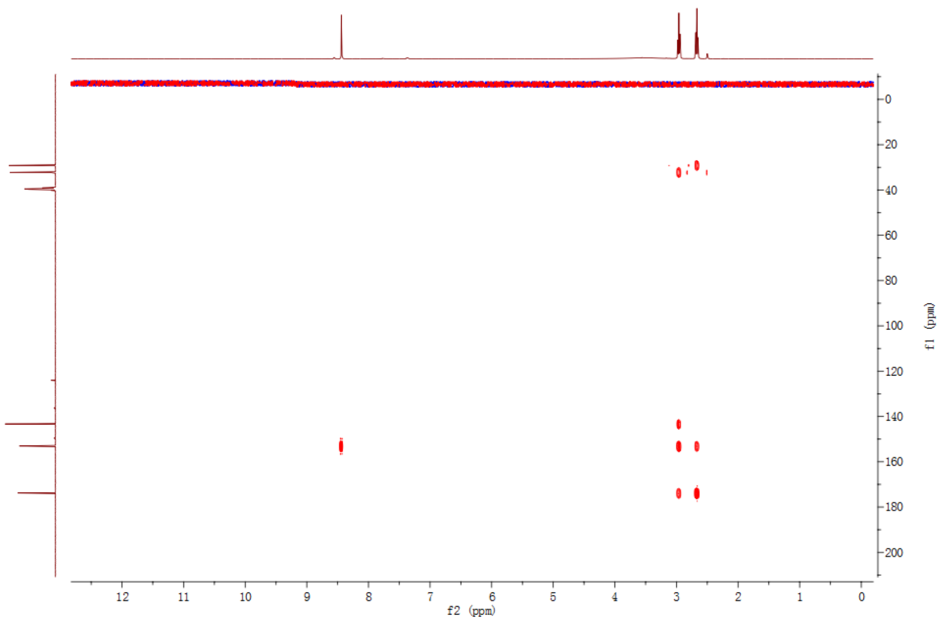

© 2014 by the authors; licensee MDPI, Basel, Switzerland. This article is an open access article distributed under the terms and conditions of the Creative Commons Attribution license (http://creativecommons.org/licenses/by/4.0/).
